# *In Utero* Alcohol Exposure Impairs Retinal Angiogenesis and the Microvessel-Associated Positioning of Calretinin Interneurons

**DOI:** 10.1523/ENEURO.0295-22.2022

**Published:** 2023-04-25

**Authors:** Marion Dumanoir, Anaïs Leroy, Delphine Burel, Annie Laquerrière, François Janin, Alexis Lebon, Manon Valet, David Godefroy, Lauriane Przegralek, Maryline Lecointre, Serge Picaud, Stéphane Marret, Florent Marguet, Bruno J. Gonzalez, Carole Brasse-Lagnel

**Affiliations:** 1 Rouen Université Institut National de la Santé et de la Recherche Médicale Unité 1245, Genomic and Personalized Medicine in Cancer and Neurological Disorders, Institute for Research and Innovation in Biomedicine (IRIB), Normandie University, 76183, Rouen, France; 2 Regional Platform for Cell Imaging of Normandy (PRIMACEN), Institute for Research and Innovation in Biomedicine (IRIB), Rouen University, 76183, Rouen, France; 3 Department of Pathology, Rouen University Hospital, 76183, Rouen, France; 4 Institut National de la Santé et de la Recherche Médicale Unité 1096, Laboratory EnVI, Normandie University, Rouen University, 76183, Rouen, France; 5 Rouen Université, Institut National de la Santé et de la Recherche Médicale Unité 1239, Neuronal and Neuroendocrine Differentiation and Communication Laboratory, Institute for Research and Innovation in Biomedicine (IRIB), Normandie University, Rouen University, 76183, Rouen, France; 6 Institut National de la Santé et de la Recherche Médicale, Centre National de la Recherche Scientifique, Institut de la Vision, Sorbonne Université, 75006, Paris, France; 7 Department of Neonatal Paediatrics and Intensive Care and Neuropaediatrics, Rouen University Hospital, 76183, Rouen, France; 8 Department of Biochemistry, Rouen University Hospital, 76183, Rouen, France

**Keywords:** alcohol, development, neonate, neurovascular, retina, vessel

## Abstract

In addition to brain disorders, which constitute a devastating consequence of prenatal alcohol exposure (PAE), eye development is also significantly affected. Given that the retina is a readily accessible part of the central nervous system, a better understanding of the impact of ethanol on retinal development might provide ophthalmological landmarks helpful for early diagnosis of fetal alcohol syndrome. This study aimed to provide a fine morphometric and cellular characterization of the development of retinal microvasculature and neurovascular interactions in a mouse model of fetal alcohol spectrum disorder (FASD). The data revealed that PAE impaired superficial vascular plexus development. In particular, progression of the vascular migration front was significantly decreased in PAE retinas, supporting a delay in plexus progression. Moreover, a significant decrease in the vessel density and number of perforating vessels was quantified in PAE mice, supporting less angiogenesis. The present study provides also the first evidence of a close interaction between migrating calretinin-positive interneurons and perforating microvessels in the inner nuclear layer of the developing retina. This neurovascular association was significantly impaired by PAE. Moreover, projections of amacrine cells were abnormally distributed and densified in stratum S1 and S2. In humans, comparison of a five-month-old control infant with a three-month-old alcohol-exposed case revealed a similar mispositioning of calretinin-positive interneurons. This opens new research avenues regarding a neurovascular contribution in the deleterious effects of alcohol in the developing retina and support that ophthalmological examination could become a promising approach for early detection of alcohol-exposed infants presenting with neurovascular brain defects.

## Visual Abstract

**Figure:**
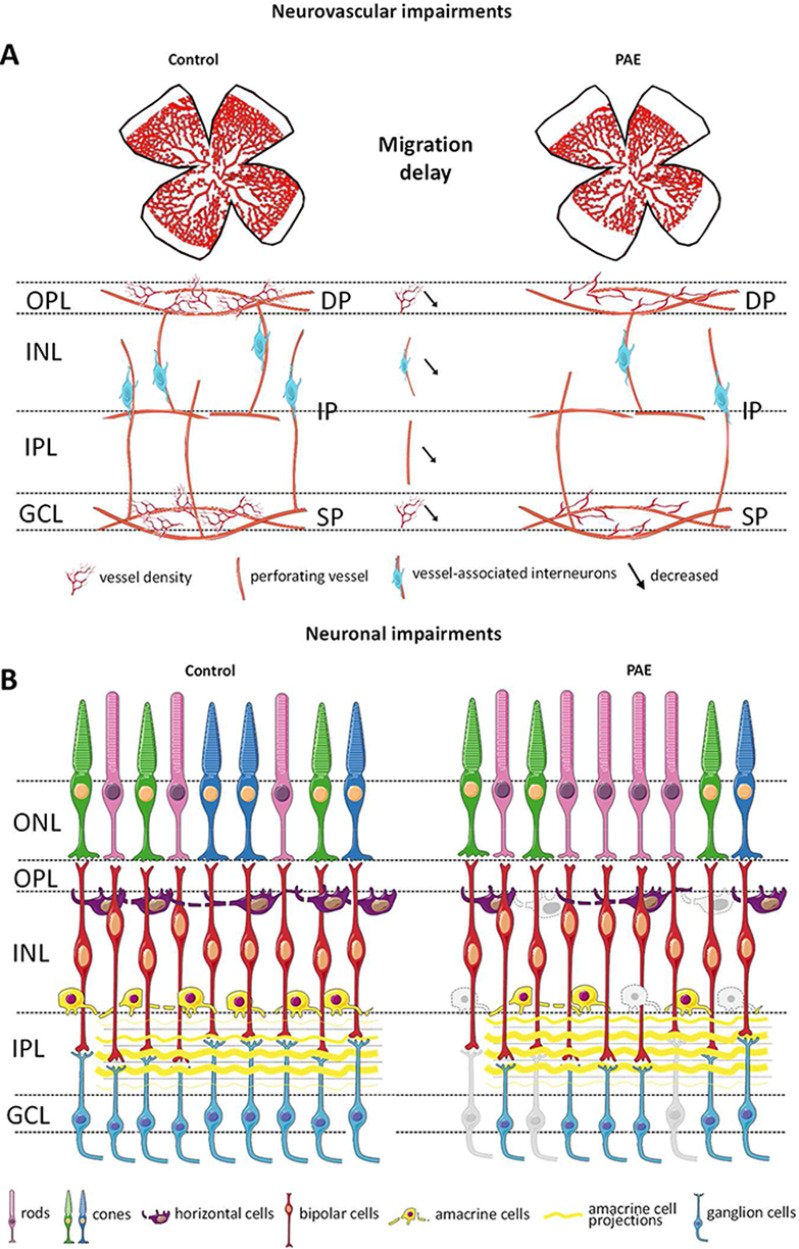

## Significance Statement

In the developing brain, prenatal alcohol exposure (PAE) has been shown to disorganize cortical vasculature leading to defective layering of interneurons which use radial microvessels to enter the neocortex. Using a murine model of fetal alcohol spectrum disorder (FASD), here, we show that PAE impairs the retinal microvasculature development and neuronal organization. In particular, migrating calretinin-positive interneurons of the internal nuclear layer are associated with perforating microvessels. PAE reduces the number of vessel-associated calretininergic cells and impairs their positioning. The mispositioning of calretinin-positive interneurons is also observed in a human FAS infant. These findings provide new evidence that PAE-induced cortical impairments are found in retina. They offer promising tools for the early diagnosis of brain impairments in infants *in utero* exposed to alcohol.

## Introduction

Prenatal alcohol exposure (PAE) is a main cause of neurodevelopmental outcomes worldwide and the most prevalent cause in industrialized countries (Bakoyiannis et al., 2014; Popova et al., 2017). Global prevalence of fetal alcohol spectrum disorder (FASD) among children and youth in the world population is estimated to be 7.7 per 1000 people with marked disparities between countries (Lange et al., 2017; Popova et al., 2017). Fetal alcohol syndrome (FAS) is the most severe expression of FASD and is characterized by cranio-facial dysmorphisms, growth retardation, and cognitive disabilities (Wozniak et al., 2019). At birth, FAS diagnosis is based on characteristic facial features. However, PAE causes a continuum of disabilities, and many FASD children do not exhibit the characteristic physical features of FAS (Memo et al., 2013; Wozniak et al., 2019). Consequently, while infants with FASD will progressively express neurobehavioral disabilities, they frequently remain undetected until school age (Chasnoff et al., 2015).

Neurobehavioral disabilities because of FASD are the consequence of neurodevelopmental insults, and several studies using MRI have demonstrated numerous various structural brain alterations, such as reduced volumes of cortical gray matter, corpus callosum, or cerebellum (Treit et al., 2013; Inkelis et al., 2020). However, these investigations were performed in already diagnosed FAS children or retrospectively at ages ranging from five years to adulthood when neurobehavioral deficits had already been detected. Thus, the early diagnosis of before the age of three, i.e., before the onset of neurodevelopmental disorders, is challenging for clinicians to start clinical care when brain plasticity is maximal (Lerna et al., 2014).

Most studies using preclinical models first focused on nerve cells. For example, PAE impairs neuron differentiation and plasticity and promotes cell death (Olney, 2004). Similarly, regarding glial cells, ethanol inhibits astrocyte-mediated neuritogenesis as well as oligodendrocyte survival and myelination (Guizzetti et al., 2014). In 2012, Jégou and coworkers provided the first evidence that PAE also affects angiogenesis in the developing cortex (Jégou et al., 2012). During brain development, angiogenesis and neurogenesis are concomitant events (Carmeliet and Tessier-Lavigne, 2005). In particular, two nervous cell types, GABAergic interneurons (Won et al., 2013) and oligodendrocytes (Tsai et al., 2016), require vessels as guides during their migration. Recent data from our research group showed a disruption of the vessel-associated migration of GABAergic interneurons (Léger et al., 2020a,b; Marguet et al., 2020). Nevertheless, even if intracortical microvasculature disorganisation resulting from *in utero* alcohol exposure could be a promising tool for the early diagnosis of FASD, access to brain microvessels by means of MRI remains challenging in human neonates.

The retina is part of the central nervous system, and eye fundus acquisition is a rapid, slight, and economical clinical act currently used to diagnose retinopathies of preterm neonates (Sapieha et al., 2010). PAE has been shown to induce visual system alterations (Strömland, 1987; Chan et al., 1991; Strömland and Pinazo-Durán, 2002), which persist until adulthood (Gyllencreutz et al., 2020). For example, in a cohort of Swedish children with FAS, fundus analysis revealed tortuosity of large vessels and optic nerve hypoplasia with deleterious consequences to the visual function of children (Strömland, 1982; Pinazo-Duran et al., 1997; Hellström, 1999). However, to our knowledge, retinal angiogenic and neurovascular defects similar to those observed in the developing neocortex have not been reported in the literature (Léger et al., 2020b; Marguet et al., 2020).

Considering the recent major advances supporting a functional link between microvasculature defects and neurodevelopmental impairments (Won et al., 2013; Léger et al., 2020a), we hypothesized that: (1) PAE could alter the development of the retinal microvasculature, (2) retinal developmental defects could mirror the alcohol-induced neurovascular disorders recently demonstrated in the developing cortex and, consequently, (3) a precise characterization of neurovascular defects in alcohol-exposed retinas could provide new perspectives for the early diagnosis of FASD neonates devoid of facial dysmorphisms. Using a mouse model of FASD, the aim of the present study was to characterize the effect of PAE on microvasculature development and neuronal defects in the retina.

## Materials and Methods

### Animals

NMRI (Naval Medical Research Institute) mice were purchased from Janvier and reared in controlled temperature rooms (21 ± 1°C) with a 12/12 h light/dark cycle with free access to food and water. Animal manipulations were performed according to the recommendations of the European Communities Council Directives (86/609/EEC) and the French National legislation (ethical approval no. 01680.02) and were supervised by authorized investigators (BJG, authorization no. 7687 from the French Ministry of Agriculture and Fisheries).

### *In vivo* treatment of pregnant mice and newborns

Pregnant NMRI mice received a daily subcutaneous injection of sodium chloride (0.9% NaCl) or ethanol (3 g/kg) diluted (50% v/v) in 0.9% NaCl, as previously described (Jégou et al., 2012). Injections were performed from gestational day (GD)15 to GD19. The volume of injections was recalculated each day of treatment according to the weight of the pregnant mouse. Mothers received a single injection of the same treatment at parturition [postnatal day (P)0] and 1 d after parturition (P1). The experiments were done at different developmental stages ranging from P2 to P15 and pups were randomly collected to maintain a uniform number of pups between litters. A total of 26 pregnant dams and litters were used in the study (12 for the control group and 14 for the PAE group). Male and female pups from randomized pregnant mice were used for the experiments.

### Whole-mount retina immunohistochemistry

Mice from the control and alcohol-exposed groups were killed at different postnatal stages [P2, P5, P10, P15, and adult (P45)] by a bolus of isoflurane (5%). Eyes were collected, fixed for 30 min in 4% paraformaldehyde (PFA) at room temperature, and washed for 15 min in PBS. Before immunostaining, retinas were isolated from the eyes by dissecting away the cornea, lens, iris, and choroid. When present, the hyaloid vasculature was also removed. Retinas were blocked and permeabilized with 0.2% bovine serum albumin (BSA) and 0.5% Triton X-100 in PBS. Blood vessels were identified with rat CD31 antibody (1:200) incubated overnight at 4°C in 0.2% BSA in PBS and then 2 h at room temperature with Alexa Fluor 594 donkey anti-rat IgG (1:400 in 0.2% BSA in PBS; Table 1). After immunostaining, retinas were cut four times to allow flower-like unfolding and flattening and were mounted with Permafluor reagent (Microm). Multiframe acquisitions of the whole retina were performed using an SP8 confocal microscope (Leica) or a THUNDER Imaging Station (Leica), using the mosaic function. Practically, a tile scan was performed with a 20× Plan Apo 0.8 NA (numerical aperture) objective at three fluorescent channels and a 15-μm Z-stack. Background correction was applied using the Instant Computational Clearing (ICC) function of the THUNDER imaging station.

**Table 1:** Antibodies

Primary antibodies (dilution)	Provider/reference	Secondary antibody (dilution)	Provider/reference
Immunochemistry			
Rat anti-PECAM/CD31 (1:200)	BD Pharmingen/550274	Donkey Alexa Fluor 594 anti-rat IgG (1:400)	Molecular Probes/A21209
Rabbit anti-Calbindin (1:200)	Abcam/ab108404	Donkey Alexa Fluor 488 anti-rabbit IgG (1:400) UltraView Universal DAB Detection kit (for medical use)	Invitrogen/A-21206 Roche Diagnostic #05269806001
Rabbit anti-Calretinin (1:500)	Thermofisher Scientific/180211	Donkey Alexa Fluor 488 anti-rabbit IgG (1:400) UltraView Universal DAB Detection kit (for medical use)	Invitrogen/A-21206 For human retina Roche Diagnostic #05269806001
Mouse anti-G_0_ α (1:500)	Merck Millipore/MAB3073	Chicken Alexa Fluor 488 anti-mouse IgG (1:400)	Invitrogen/A-21200
Rabbit anti-RBPMS (1:500)	Merck Millipore/ABN1362	Donkey Alexa Fluor 488 anti-rabbit IgG (1:400)	Invitrogen/A-21206
Rabbit anti-opsin B (1:500)	Merck Millipore/AB5407	Donkey Alexa Fluor 488 anti-rabbit IgG (1:400)	Invitrogen/A-21206
Rabbit anti-opsin RG (1:500)	Merck Millipore/AB5405	Donkey Alexa Fluor 488 anti-rabbit IgG (1:400)	Invitrogen/A-21206
Rabbit anti-rhodopsin (1:500)	Abcam/ab112576	Donkey Alexa Fluor 488 anti-rabbit IgG (1:400)	Invitrogen/A-21206

Western blotting			
Mouse anti-β-Actin (1:5000)	Sigma–Aldrich/A5441	Donkey anti-mouse HRP (1:5000)	Jackson ImmunoResearch/715-035-151
Goat anti-PECAM/CD31 (1:1000)	Santa Cruz/sc-1506	Rabbit anti-goat HRP (1:5000)	Jackson ImmunoResearch/305-035-045

Clearing			
Goat Anti podocalyxin (1:200)	RD system/AF1556	Donkey Cy3TM anti-goat (1:400)	Jackson ImmunoResearch/705-165-147
Rat anti-PECAM/CD31 (1:200)	BD Pharmingen/550274	Donkey Alexa 594 anti-rat (1:200)	Molecular Probes/A21209
Mouse anti-α Sma-Cy3 (1:400)	Sigma/C6198	/	/
Rabbit anti-Calretinin (1:400)	Thermofisher Scientific/180211	Donkey Cy5TM anti-rabbit (1:200)	Jackson ImmunoResearch/711-175-152

### Morphometric analysis of whole-mount retinas

According to Morita and coworkers, regions of interest (ROIs) were defined in the central and peripheral parts of retinas, and vessel density, as well as several additional indicators of microvascular development, were quantified by using the angiogenesis application from ImageJ software (National Institutes of Health; Morita et al., 2017). In particular, the following were measured: (1) distance remaining to be covered by the superficial vascular plexus (distance between the vascular migration front and the retinal edge), (2) vessel density measured by thresholding of CD31-positive structures, (3) mesh number, (4) mean segment length of meshes (total segment length/number of segments), (5) large vessel tortuosity index (measured vessel length/straight‐line vessel), and (6) arteriovenous crossovers (a crossover was identified when an artery crossed a vein). Each parameter was measured in four areas per retina.

### Cross sections of retina

Enucleated eyes were fixed in 4% PFA for 1 h and subsequently incubated for 12 h in PBS/sucrose 10%, 20%, and 30%. Eyes were then included in NEG-50 (Richard-Allan Scientific, 6502) in an orientation that facilitated sectioning in transverse planes and frozen in liquid nitrogen embedded. Ice-cooled NEG-50 tissue blocks were sectioned to generate serial slices (20 μm) using a cryostat (Leica CM1950). Sections including the optic nerve head were subsequently used for immunohistochemical labeling and were postfixed for 30 min, incubated in blocking solution (3% BSA in PBS Tween 20 0.1%) for 1 h, and then incubated overnight in the same solution containing a primary antibody (Table 1). Then, slices were rinsed twice with PBS for 20 min and incubated for 2 h at room temperature within the same incubation buffer containing the appropriate secondary antibody (Table 1). Before mounting within Permafluor medium (Microm), cell nuclei were labeled by incubating the slices for 5 min with 1 μg/ml Hoechst 33258 in PBS.

Primary antibodies used for immunohistochemistry in cross sections of retinas were as follows: Rat anti-PECAM/CD31 (BD PharMingen, 550274, 1:200), Rabbit anti-Calbindin (Abcam, ab108404, 1:200), Rabbit anti-Calretinin (Thermofisher Scientific, 180211, 1:500), Mouse anti-G0 α (Merck Millipore, MAB3073, 1:500), Rabbit anti-RBPMS (Merck Millipore, ABN1362, 1:500), Rabbit anti-opsin B (Merck Millipore, AB5407, 1:500), Rabbit anti-opsin RG (Merck Millipore, AB5405, 1:500), and Rabbit anti-rhodopsin (Abcam, ab112576, 1:500). Secondary antibodies used were as follows: Donkey Alexa Fluor 594 anti-rat IgG (Invitrogen, A21209, 1:400), Donkey Alexa Fluor 488 anti-rabbit IgG (Invitrogen, A-21206, 1:400), Chicken Alexa Fluor 488 anti-mouse IgG (Invitrogen, A-21200, 1:400).

### Cross-section image analysis

After immunolabeling, retinal sections were imaged with a THUNDER Imaging Systems or an SP8 confocal microscope, and the number of positive cells within designated areas was measured using ImageJ software. Analyses were focused on the central retina defined as the retina section near the optic nerve and on the periphery of retina corresponding to the analysis of the ventral and the dorsal regions of retina. At the periphery results were expressed as the average of ventral and dorsal regions. For each region, four ROIs of similar sizes were defined. For each ROI, the number of positive cells were summed, and the total number was divided by the total length (μm) in which cells were counted. The result was reported as the number of positive cells/100 μm for each marker. The density of microvessel and rhodopsin-positive cells was expressed as the proportion of the total surface of the positively stained area divided by the total area. Thicknesses of whole retina and distinct neuronal layers, i.e., retinal neuroblastic layer (RNL), inner plexiform layer (IPL), inner nuclear layer (INL), outer plexiform layer (OPL), and outer nuclear layer (ONL) were measured by using ImageJ software. For each animal, results were expressed as the mean of the four ROIs and replicated in several independent animals per group ranging from five to seven regarding a given experiment.

### Quantification of amacrine cell projections in the IPL

Measurements of calretinin-positive cell projections in the IPL were performed after immunostaining of P15 slices with a calretinin antibody (Table 1). After 10× magnification acquisitions, quantification of calretinin fluorescent intensity profiles in the developing retina was performed in animals prenatally exposed to NaCl or ethanol using the “line scan” tool of the MetaMorph software (Roper Scientific). Then, the area under the curve of retinal strata (S1–S5) was evaluated.

### Tissue clearing

Eyes were fixed in 4% PFA overnight at 4°C and washed with 1× PBS three times for 10 min. The eyes were then dehydrated in increasing concentrations of methanol (20%, 40%, 60%, 80%, and 100% methanol, 0.1% PBS) for 1 h each at room temperature. After bleaching in chilled fresh 5% H2O2 in methanol overnight at 4°C, the samples were rehydrated in decreasing baths of methanol (80%, 60%, 40%, and 20% of methanol in PBS 1×) for 1 h each and washed twice for 1 h in a PBS 1× solution. The eyes were then successively incubated for 1 d at 37°C with a permeabilization solution (20% DMSO, 23 mg/ml glycine, 0.1 g/l thimerosal, and 0.2% Triton X-100) and with a blocking solution (6% normal donkey serum, 10% DMSO, and 0.1 g/l thimerosal). For immunostaining, primary antibodies (anti-CD31, anti-podocalyxin, anti-α Sma, and anti-calretinin antibodies (Table 1) were added to a solution containing 5% DMSO, 3% NDS, 10 mg/ml heparin, and 0.1 g/l thimerosal in 1× PBS for 6 d at 37°C. The samples were washed 6 times for 1 h in the same solution without antibody. Donkey anti-goat secondary antibodies coupled with Cy3TM (705–165-147; Jackson ImmunoResearch), Donkey anti-rat Alexa 594 (A21209; Invitrogen) and Donkey anti-rabbit Cy5 (711-175-152; Jackson ImmunoResearch; Table 1) were added for 5 d at 37°C in a solution containing 10 mg/ml heparin and 0.1 g/l thimerosal 3% NDS in 1× PBS. The samples were then washed 6 times for 1 h at 37°C in the same solution without antibodies before embedding in 1.5% agarose in 1× TAE. The eyes were then dehydrated in increasing methanol solutions (20%, 40%, 60%, 80%, and 100% twice of methanol in PBS 1×) for 1 h at each concentration at room temperature and placed in a solution containing 33% methanol and 66% dichloromethane (DCM) overnight at room temperature with shaking. The last step consisted of 2 baths of 100% DCM for 15 min under shaking. Finally, the eyes were cleared in di-benzyl ether (DBE) until imaging. 3D imaging was performed using an ultramicroscope II, LaVision BioTec, and analyzed with IMARIS software.

### RT-PCR

Expression of the endothelial gene CD31 was quantified by qRT–PCR. RNA was extracted from dissected retinas using a Nucleospin RNA kit (Macherey-Nagel) according to the manufacturer’s instructions, and cDNA was synthetized using an Im-Prom II Reverse Transcriptase kit (Promega). The expression of CD31 was analyzed using forward and reverse primers (F-TCCAACAGAGCCAGCAGTATGAG; R-TCCAATGACAACCACCGCAATG) combined with SYBR Green Master Mix (Thermo Fisher Scientific) in 384-well plates with a Bravo liquid handling system (Agilent). PCR amplification was conducted using a QuantStudio 12K Flex (Thermo Fisher Scientific). Each replicate cycle threshold (Ct) was normalized to the Ct of glyceraldehyde-3-phosphate dehydrogenase (GAPDH), which was used as an endogenous control gene (primer forward: F-CATGGCCTTCCGTGTTCCTA; primer reverse: R-CCTGCTTCACCACCTTCTTGA). Changes in gene expression were compared with the mean of the control group by using the 2^-ΔΔCt^ method (Livak et al., 2001) and expressed as fold change.

### Western blotting

Protein content of dissected retina was extracted in lysis buffer [50 mm HEPES, 150 mm NaCl, 10 mm EDTA, 10 mm glycerophosphate, 100 mm NaF, 0.25% NP40, 1 mm PMSF, protease inhibitor cocktail (P8340, Sigma-Aldrich), and phosphatase inhibitor cocktail 2 and 3 (P5726 and P0044, Sigma-Aldrich)]. After centrifugation (20 min at 12 000×g), the supernatant was saved as protein extract. Proteins (50 μg) were loaded onto SDS–PAGE gels containing 7.5% acrylamide. After migration, proteins were blotted onto Protran nitrocellulose membranes (10600003, GE Healthcare). The membranes were then blocked by incubation for 1 h with 5% BSA/TBS Tween 20 (0.1%) and incubated overnight at 4°C with the primary antibody (anti-PECAM/CD31; 1:1000). After incubation with the appropriate secondary antibody coupled to horseradish peroxidase (Rabbit anti-goat HRP; Table 1), proteins were visualized by chemiluminescence using the ECL plus immunoblotting detection kit (GE Healthcare Europe GmbH). Equal protein loading was checked by stripping the membranes and reprobing them with β-actin antibody (1:5000, A55441, Sigma-Aldrich). The intensity of the immunoreactive bands was quantified by densitometry with Image Lab 5.0 software from Bio-Rad, and the results are expressed as a ratio with respect to β-actin immunoreactivity intensity. Commercial markers (Seeblue prestained standard, Invitrogen) were used as molecular weight standards.

### Description of human cases

The FAS was the second child born to nonconsanguineous parents. He was delivered at 38 WG. His birth weight was 2260 g (<5th percentile). He presented craniofacial dysmorphism characteristic of FAS associated with short palpebral fissures, smooth philtrum, and thin vermillion border because of prenatal alcohol exposure, which had been self-reported by the mother. Postnatal life was uneventful until three months of age, when he died of sudden infant death syndrome. A complete autopsy was performed, confirming craniofacial dysmorphism and growth retardation. Macroscopic and histologic examination of all viscera did not reveal any lesions. Examination of the brain revealed cerebral edema, but no other macroscopic or microscopic lesions were found. During autopsy, the eyes were removed, fixed in a Bouin fixative solution, embedded in paraffin, and 6-μm sections were stained with hematoxylin-eosin.

A control case was selected for comparison. This child was born at 37 WG. Postnatal course was normal until five months of age, when he died from unexpected death syndrome. He was discovered on his back in his bed by his mother. Pathologic examination of the viscera made it possible to discover pyelonephritis with perirenal phlegmon of the right kidney. The pathologic report concluded a fatal toxic-infectious shock of sudden onset. Associated lesions consisted of histologic signs of acute liver failure with subsequent diffuse cortical cerebral edema. As in the latter case, the eyes were removed and processed according to the same protocol.

In both cases, the parents gave their written informed consent for autopsies, which were performed in accordance with French law.

### Immunohistochemistry in human retinas

Immunohistochemical visualization of calbindin-positive or calretinin-positive cells was conducted on 6-μm sections obtained from paraffin-embedded material according to standardized protocols using antibodies listed in Table 1. Before incubation with the primary antibodies as described above in the paragraph “whole-mount retina immunohistochemistry,” sections were deparaffinized and submitted to a microwave pretreatment protocol to aid antigen retrieval. After incubation, the slides were processed using the Ultraview Universal DAB detection kit (Roche Diagnostic #05269806001) coupled to the Ventana medical system (Table 1).

### Statistical analysis

Statistical analyses were performed using Graph Pad Prism 6.01. The results are presented as boxes and whiskers corresponding to the median with the minimum and maximum, and the Mann–Whitney *U* test was used to compare the medians between PAE and age-matched control groups. Statistically significant differences between multiple comparisons were determined using a two-way ANOVA with Tukey’s *post hoc* test. Significance was defined by *p values < 0.05.

## Results

### PAE impaired the microvascular network of the retinal superficial plexus

**Figure 1.**
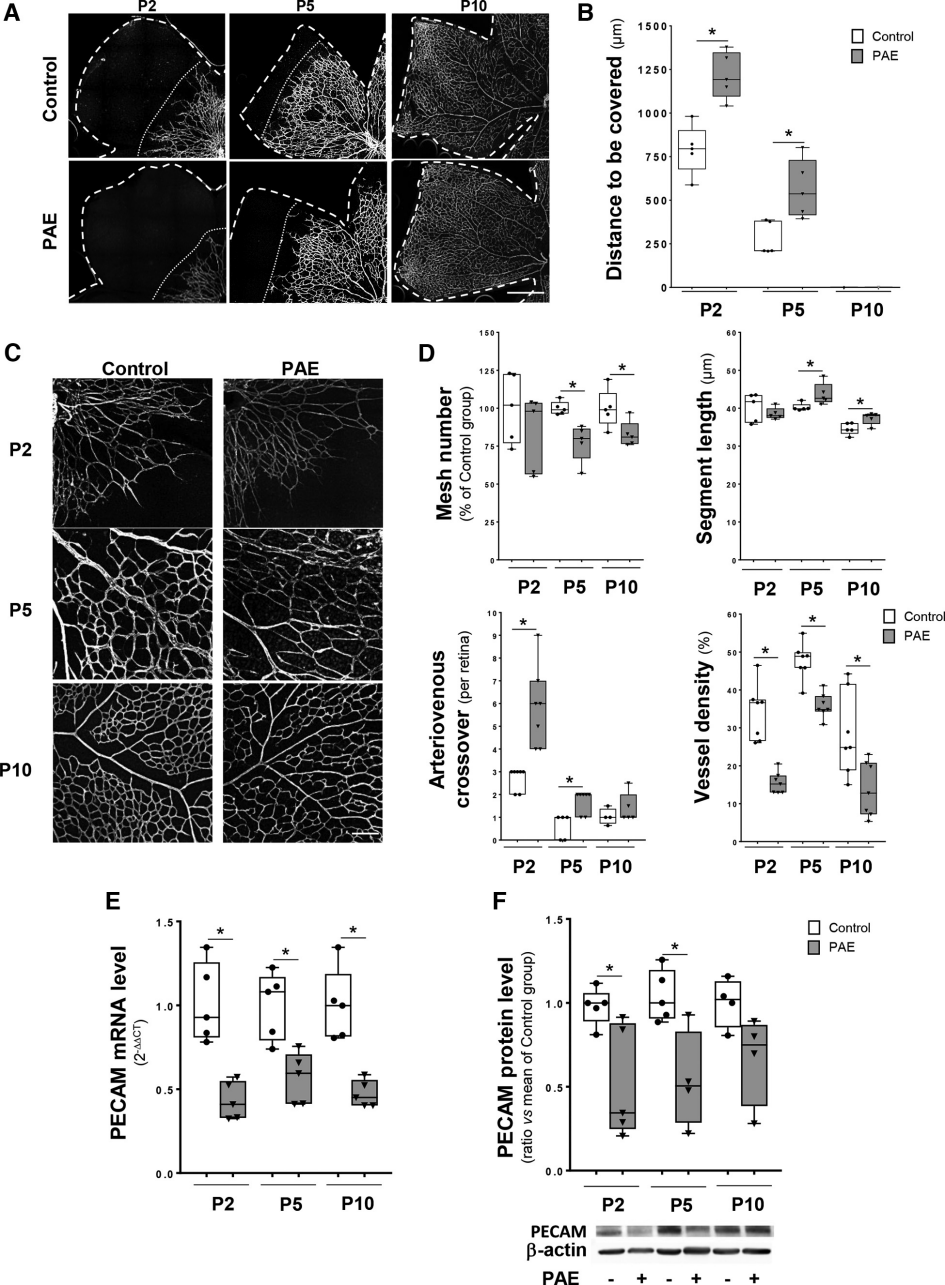
Effect of PAE on the development of the superficial vascular plexus from P2 to P10. Whole-mount retinas from postnatal days (P)2, P5, and P10 were immunostained for endothelial cells using a PECAM/CD31 antibody. ***A***, Representative images obtained by THUNDER microscopy in the control and PAE groups. The scale bar represents 500 μm. ***B***, Quantification of the distances to be covered by the vascular migratory front in control (white boxes) and PAE (gray boxes) mice at P2, P5, and P10. *p < 0.05 compared versus the age-matched control group using the Mann–Whitney test, *n* = 5 pups per group. ***C***, Visualization at high magnification of the microvascular network in the middle part of retinas from the control and PAE groups at P2, P5, and P10. The scale bar represents 50 μm. ***D***, Quantification of the number of meshes, length of vessel segments, number of arteriovenous crossovers, and vessel density in retinas from the control (white boxes) and PAE (gray boxes) groups at P2, P5, and P10. *p < 0.05 compared versus the age-matched control group using the Mann–Whitney test, *n* = 5–7 pups per group. Quantification of vessel tortuosity and arteriovenous angles is shown in Extended Data Figure 1-1. ***E***, ***F***, Quantification of CD31 mRNA (***E***) and protein (***F***) levels at P2, P5, and P10 by qRT–PCR and Western blotting, respectively. *p < 0.05 compared *versus* the age-matched control group using the Mann–Whitney test, *n* = 4–5 pups per group. PAE: prenatal alcohol exposure.

Using our previously published murine model of FASD, a time course study covering the period of the superficial network development was performed to investigate whether *in utero* alcohol exposure impaired the progression of the vessel migratory front (Fig. 1*A*,*B*). In the control condition, the superficial vascular plexus progressively extended radially from the optic nerve to the retina border. This process occurs during the first 10 d after birth. Upon PAE, the distance remaining to be covered by the migratory front was significantly increased at P2 (50% increase; *p* = 0.0079, *n* = 5 for control and PAE groups, Mann–Whitney test, *U* = 0) and P5 (150% increase, *p* = 0.0079, *n* = 5 for control and PAE groups, Mann–Whitney test, *U* = 0), indicating a delayed progression of the superficial vascular plexus (Fig. 1*A*,*B*). At P10, the vascular plexus reached the retinal border, and no difference was observed between control and PAE conditions (Fig. 1*A*,*B*).

10.1523/ENEURO.0295-22.2022.f1-1 Extended Data Figure 1-1 Effect of PAE on vessel tortuosity and arteriovenous angles. Whole-mount retinas from postnatal day (P)2, P5, and P10 mice were immunostained with CD31 antibody to visualize microvessels. ***A***, Quantification of vessel tortuosity in the control and PAE groups. The inset illustrates how the ratio L2 (measured vessel length)/L1 (theoretical straight vessel) was used as an index of tortuosity. ***B***, Quantification of the arteriovenous angles in the control and PAE groups. The inset illustrates how the angle value was calculated between two large vessels originating from the optic nerve. Download Figure 1-1, TIF file.

To analyze the microvascular network of the superficial plexus, a morphometric study was performed in control and PAE retinas at P2, P5, and P10 (Fig. 1*C*,*D*). The multiparametric quantification consisted in the mesh number, vessel segment length, number of arteriovenous crossovers, and vessel density measurements (Fig. 1*D*). Two additional parameters, vessel tortuosity and arteriovenous angles, were also quantified (Extended Data Fig. 1-1). Regarding vascular meshes, no effect of PAE was found at P2, whereas ethanol significantly reduced by 20% the number of meshes at P5 and P10 (*p* = 0.0079, *U* = 0 at P5, and *p* = 0.0317, *U* = 2 at P10, *n* = 5 for all groups, Mann–Whitney test; Fig. 1*D*). The effect of PAE on mesh number was associated with a significant increase in vessel segment length at both stages (7% increase at P5, *p* = 0.0159, *U* = 1 and 11% at P10, *p* = 0.0317, *U* = 2, *n* = 5 for all groups, Mann–Whitney test; Fig. 1*D*). Moreover, at P2 and P5, arteriovenous crossovers were significantly more frequent in PAE retinal mice (2-fold increase at P2, *p* = 0.0006 *n* = 7 for control and PAE groups, *U* = 0 and 2.67-fold increase at P5, *p* = 0.0139, control *n* = 5 and PAE *n* = 7, *U* = 3, Mann–Whitney test; Fig. 1*D*). This observation was no longer visible at P10 (Fig. 1*D*). Contrasting with observations reported in FAS infants, no effect of PAE was observed on the tortuosity of large vessels (Extended Data Fig. 1-1*A*). In the same way, PAE did not modify the arteriovenous angles regardless of the developmental stage (Extended Data Fig. 1-1*B*). From P2 to P10, the vessel density of the microvascular network in the superficial plexus after PAE was drastically reduced (decrease by 59% at P2, *p* = 0.006, *U* = 0; 29% at P5 *p* = 0.0012, *U* = 1 and 49% at P10, *p* = 0.0175, *U* = 6, *n* = 7 for all groups, Mann–Whitney test; Fig. 1*D*). To corroborate these morphometric results with molecular data, the expression profile of the endothelial protein PECAM/CD31 was quantified from P2 to P10. Regardless of the stage, qRT–PCR experiments showed a strong reduction in PECAM/CD31 expression in PAE mice (decrease by 56% at P2, *p* = 0.0079, *n* = 5 for control and PAE groups, *U* = 0; 45% at P5, *p* = 0.00,159, *n* = 5 for control and PAE groups, *U* = 1 and 55% at P10, *p* = 0.00,079 *n* = 7 for control and PAE groups, *U* = 0, Mann–Whitney test; Fig. 1*E*). Similar results were obtained using Western blot analysis when considering the PECAM/CD31 protein (decrease by 66% at P2, *p* = 0.0317, *n* = 5 for control and PAE groups, *U* = 2; 50% at P5 *p* = 0.0397, control *n* = 5 and PAE *n* = 4, *U* = 1.5, Mann–Whitney test; Fig. 1*F*). Altogether, these data indicate that PAE slows down and impairs the development of the superficial vascular plexus.

### PAE impairs the microvascular network of the superficial, intermediate, and deep plexuses at P15

**Figure 2.**
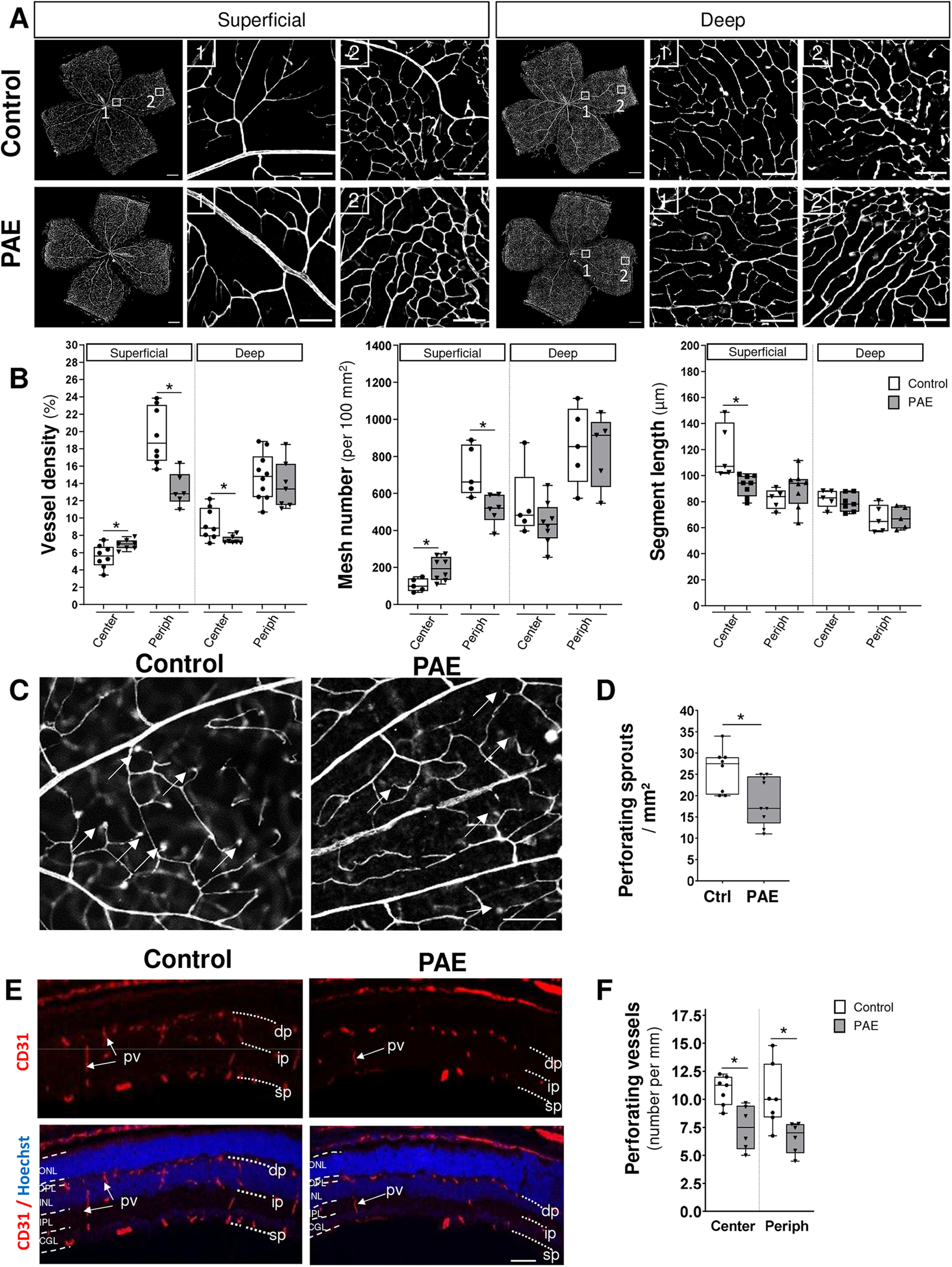
Effect of PAE on the development of the superficial and deep vascular plexuses at P15. Whole-mount retinas from postnatal days (P)15 mice were immunostained for endothelial cells using a PECAM/CD31 antibody. Quantification of intermediate and deep vessel density is shown in Extended Data Figure 2-1*A* and visualization of the superficial, intermediate, and deep vascular plexus is shown in Extended Data Figure 2-1*B*. ***A***, Visualization of the superficial and deep vascular plexus in the center (1) and at the periphery (2; Periph) parts of retinas by confocal microscopy in control and alcohol-exposed foetuses (PAE). Scale bars represent 500 and 50 μm for low and high magnifications, respectively. ***B***, Quantification of vessel density, number of meshes, and vessel segment lengths in the center and at the periphery of the superficial and deep vascular plexus from control (white boxes) and PAE (gray boxes) retinas. *p < 0.05 compared with the age-matched control group. Mann–Whitney test, *n* = 5–8 pups per group. ***C***, Visualization of perforating vessels (arrows) immunostained with a CD31 antibody in whole-mount retinas from the control and PAE groups. The scale bar represents 50 μm. ***D***, Quantification of the number of perforating sprouts in retinas from the control and PAE groups at P15. *p < 0.05 compared with the age-matched control group using the Mann–Whitney test, *n* = 8–9 pups per group. ***E***, P15 cross sections in the center part of retinas colabeled with a CD31 antibody and Hoechst in control and PAE mice. Arrows indicate perforating microvessels. The scale bar represents 100 μm. ***F***, Quantification of the number of perforating vessels in cross sections of central (Center) and peripheral (Periph) parts of retinas obtained from control and PAE mice at P15. *p < 0.05; compared with the age-matched control group using the Mann–Whitney test, *n* = 6–7 pups per group. Ctrl: Control; dp: deep vascular plexus; GCL: ganglion cell layer; INL: inner nuclear layer; ip: intermediate vascular plexus; IPL: inner plexiform layer; ONL: outer nuclear layer; OPL: outer plexiform layer; PAE: prenatal alcohol exposure; Periph: periphery; pv: perforating vessels; sp: superficial vascular plexus.

In mice, the development of the deep vascular plexus starts beyond P10 after the establishment of the superficial plexus (Stahl et al., 2010). Afterward, recurrent vessels arising from the deep plexus generate the intermediate plexus. To explore the effect of PAE on the intermediate and deep microvascular networks, we analyzed vascular plexuses on flat-mount retinas at P15 (Fig. 2; Extended Data Fig. 2-1). Quantification of the vascular density revealed that after PAE, the reduction in the microvessel density observed in the superficial plexus at P10 was also measured at P15 in the intermediate (decreased by 30%, *p* = 0.03, *n* = 7 for control group and *n* = 5 for PAE group, *U* = 3, Mann–Whitney test; Extended Data Fig. 2-1*A*) and deep vascular plexuses (decreased by 22%, *p* = 0.017, *n* = 7 for control group and *n* = 5 for PAE group, *U* = 3, Mann–Whitney test; Fig. 2*B*; Extended Data Fig. 2-1*A*).

10.1523/ENEURO.0295-22.2022.f2-1 Extended Data Figure 2-1 Superficial, intermediate, and deep vascular plexuses in retina of P15 mice. Retinas from postnatal day (P)15 mice were immunostained with CD31 antibody to visualize microvessels. ***A***, Quantification of vessel density of the intermediate and deep vascular plexus from control (white boxes) and PAE (grey boxes) groups in the center of retinas. *p < 0.05 compared with the age-matched control group. Mann–Whitney test, *n* = 5–7 pups per group. ***B***, Visualization of the superficial, intermediate, and deep vascular plexus in the center part of retinas by confocal microscopy. Scale bars represent 50 μm. Download Figure 2-1, TIF file.

Since at P15, the intermediate plexus is not yet completed (Extended Data Fig. 2-1*B*), analysis of mesh number and segment length was only conducted in the superficial and deep networks on the central and peripheral parts of the retina (Fig. 2*A*,*B*). After PAE, results obtained in the superficial plexus, revealed that vessel density was reduced especially in the peripheral retina (0.65-fold change, *p* = 0.0013, controls *n* = 8 and PAE *n* = 6, *U* = 1, Mann–Whitney test; Fig. 2*B*). In contrast, in the central retina, vessel density (1.20-fold change, *p* = 0.040, controls *n* = 8 and PAE *n* = 7, *U* = 10, Mann–Whitney test; Fig. 2*B*) and mesh number (increase by 97%, *p* = 0.02, controls *n* = 5 and PAE *n* = 8, *U* = 5, Mann–Whitney test; Fig. 2*B*) were increased and associated with smaller segment lengths (decrease by 12%, *p* = 0.001, controls *n* = 5 and PAE *n* = 8, *U* = 0, Mann–Whitney test; Fig. 2*B*). The same analysis was performed in the deep vascular plexus. However, the deep vessel density was slightly decreased by ∼20% in the central part of the retina after PAE (*p* = 0.028, controls *n* = 8 and PAE *n* = 7, *U* = 9, Mann–Whitney test; Fig. 2*B*). No effect of PAE was observed at the periphery of the deep vascular plexus (Fig. 2*B*). These data indicate that impairments of the superficial plexus identified at P10 are still observed at P15 and that the deep vascular plexus, although affected, is especially impacted by PAE in the central part of the retina.

### PAE impairs perforating radial vessels arising from the superficial plexus

The effect of PAE on perforating vessels (pv) was analyzed at P15 (1) on flat-mount retinas by quantifying the density of deep vessel ends (Fig. 2*C*,*D*, arrows) and (2) on transverse sections by quantifying radial vessels (Fig. 2*E*,*F*, arrows). In flat-mount retinas, PAE significantly decreased the density of vascular ends corresponding to perforating vessels (decrease by 38%, *p* = 0.019, controls *n* = 8 and PAE *n* = 9, *U* = 12, Mann–Whitney test; Fig. 2*D*). In transverse slices, the superficial (sp), intermediate (ip), and deep plexuses (dp) were visualized by PECAM/CD31 immunohistochemistry (Fig. 2*E*, upper panels, dotted lines). Morphometric analysis showed that PAE decreased the density of perforating radial vessels connecting the superficial and deep vascular plexuses, in the central (decrease by 33%, *p* = 0.008, controls *n* = 7 and PAE *n* = 6, *U* = 3, Mann–Whitney test; Fig. 2*E*, arrows, *F*) as well as in the peripheral part of retina (decrease by 26%, *p* = 0.02, Controls *n* = 8 and PAE *n* = 7, *U* = 8, Mann–Whitney test; Fig. 2*F*). These results indicate that the deleterious effect of PAE initially identified in the superficial plexus was associated with altered perforating vessels, which are subsequently involved in the development of deep and intermediate plexuses.

### PAE prevents neurogenesis in the retina

**Figure 3.**
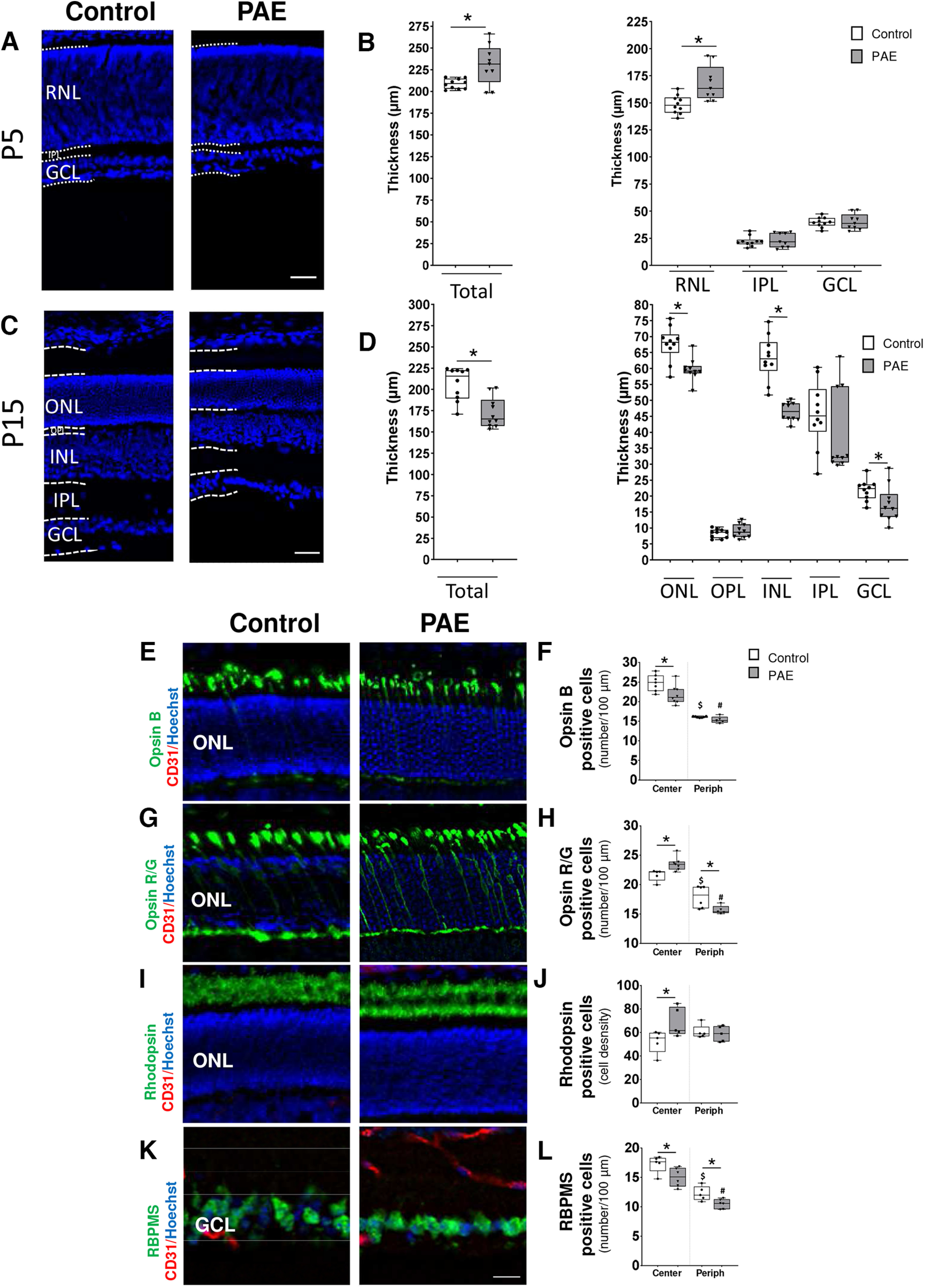
Effect of PAE on retinal layering and neuronal homeostasis. ***A–D***, High magnification of cross sections of postnatal days (P)5 and P15 mice after Hoechst labeling. Visualization of the RNL, ONL, OPL, INL, IPL, and GCL at P5 (***A***) P15 (***C***) and quantification of the whole retina thickness and distinct retinal layers (RNL, ONL, OPL, INL, IPL, and GCL) in the control (white boxes) and PAE (gray boxes) groups at P5 (***B***) and P15 (***D***). *p < 0.05 compared with the age-matched control group using the Mann–Whitney test, *n* = 9–10 pups per group. ***E–H***, Effect of PAE on cone cells at P15. Triple fluorescent labeling visualizing cone cells, vessels, and nuclei labeled by anti-opsin blue (opsin B; ***E***, ***F***) or anti-opsin red/green (opsin R/G; ***G***, ***H***), CD31 antibodies and Hoechst. Acquisitions were performed at P15 in the photoreceptor layer in the control and PAE groups. The scale bar represents 25 μm. In the central (Center) and the peripheral (Periph) parts of retinas, quantification of opsin B-positive cells (***F***) or opsin R/G-positive cells (***H***) per 100 μm in photoreceptor layers in the control (white boxes) and PAE (gray boxes) groups, *p < 0.05 versus the age-matched control using the Mann–Whitney test, *n* = 5–7 pups per group. ***I***, ***J***, Effect of PAE on rod cells at P15. Triple fluorescent labeling visualizing rod cells, vessels and nuclei labeled by anti-rhodopsin, CD31, and Hoechst, respectively. In the central (Center) and the peripheral (Periph) parts of retinas, the relative density of rhodopsin-positive cells (***J***) is expressed as a percent of the area positively stained versus the analyzed area in the photoreceptor layer in the control (white boxes) and PAE (gray boxes) groups. *p < 0.05 versus the age-matched control using the Mann–Whitney test, *n* = 5 pups per group. ***K***, ***L***, Effect of PAE on ganglion cells at P15. Triple fluorescent labeling visualizing ganglion cells, vessels, and nuclei labeled, respectively labeled by RBPMS, CD31 antibodies, and Hoechst. Quantification in central (Center) and peripheral (Periph) parts of retinas of RBPMS-positive cells per 100 μm (***L***) in the GCL in the control and PAE groups. The scale bar represents 25 μm. *p < 0.05 versus the age-matched control using the Mann–Whitney test, *n* = 5–6 pups per group. Ctrl: Control; GCL: ganglion cell layer; INL: inner nuclear layer; IPL: inner plexiform layer; ONL: outer nuclear layer; OPL: outer plexiform layer; PAE: prenatal alcohol exposure; RNL: retinal neuroblastic layer.

It is now well established that angiogenesis is concomitant with neurogenesis and that these two processes functionally interact (Carmeliet and Tessier-Lavigne, 2005). Based on the present data, which revealed a deleterious effect of PAE on retinal microvasculature, we explored alcohol-associated neurodevelopmental defects. A morphometric analysis was first performed at two developmental stages, P5 and P15 (Fig. 3*A–D*). At P5, a slight increase of 10% in retinal thickness was measured in PAE mice with a thicker neuroblastic layer (*p* = 0.0435, controls *n* = 10 and PAE *n* = 9, *U* = 20, Mann–Whitney test; Fig. 3*A*,*B*). At P15, a significant 17% reduction of the retinal thickness was measured in PAE mice compared with the control group (*p* = 0.0015, *n* = 10 for controls and PAE groups, *U* = 10, Mann–Whitney test; Fig. 3*C*,*D*). PAE induced a significant reduction in the outer nuclear (ONL; 13% decrease, *p* = 0.0027, *n* = 10 for controls and PAE groups, *U* = 12, Mann–Whitney test), inner nuclear (INL; 26% decrease, *p* < 0.0001, n= for controls and PAE groups, *U* = 0, Mann–Whitney test), and ganglionic cell layers (GCL; 28% decrease, *p* = 0.037, *n* = 10 for controls and PAE groups, *U* = 22.5, Mann–Whitney test; Fig. 3*D*). No effect was observed in the outer plexiform (OPL) and inner plexiform (IPL) layers.

To study PAE effects on retinal layer neuron positioning, we performed a precise morphometric analysis targeting distinct neuronal cell populations, i.e., cone and rod photoreceptors and ganglion, bipolar, horizontal, and amacrine cells, at P15.

### PAE impedes the normal positioning and density of photoreceptors and ganglion cells

Immunostainings of the of the retina with opsin-blue (opsin-B), opsin Red/Green (opsin-R/G), or rhodopsin antibodies displayed immunoreactivity in the outermost layer (Fig. 3*E–J*) corresponding to the photoreceptor layer. Especially in the central part of retina, a slight reduction of 10% in opsin-B-positive cell number was measured in PAE retinas (*p* = 0.028, controls *n* = 7 and PAE *n* = 6, *U* = 9, Mann–Whitney test; Fig. 3*E*,*F*), associated to an increase of 10% in opsin-R/G cell number by comparison with control retinas (*p* = 0.017, controls *n* = 5 and PAE *n* = 7, *U* = 3, Mann–Whitney test; Fig. 3*G*,*H*). In contrast, in the periphery, a decrease of 16% in opsin R/G positive cells was measured (periph; *p* = 0.017, controls *n* = 6 and PAE *n* = 5, *U* = 2, Mann–Whitney test; Fig. 3*H*). Intense rod cell immunolabeling was achieved with the antibody rhodopsin, and a significant 1.30-fold increase in PAE retinas was observed compared with controls (*p* = 0.031, *n* = 5 for control and PAE groups, *U* = 2, Mann–Whitney test; Fig. 3*I*,*J*) and this effect was not observed in the peripheral part of retina. RBPMS immunostaining revealed that ganglion cells were adequately positioned in the GCL, and the quantification of RBMPS-positive cells revealed a significant 0.87-fold decrease in ganglion cell density on PAE in the central retina (*p* = 0.030, controls *n* = 5 and PAE *n* = 6, *U* = 3, Mann–Whitney test; Fig. 3*K*,*L*). This effect was also observed in the peripheral part of retina with a significant 0.88-fold decrease in RBPMS positive cells (*p* = 0.017, controls *n* = 5 and PAE *n* = 6, *U* = 2, Mann–Whitney test; Fig. 3*K*,*L*).

### PAE alters the positioning and density of interneurons

**Figure 4.**
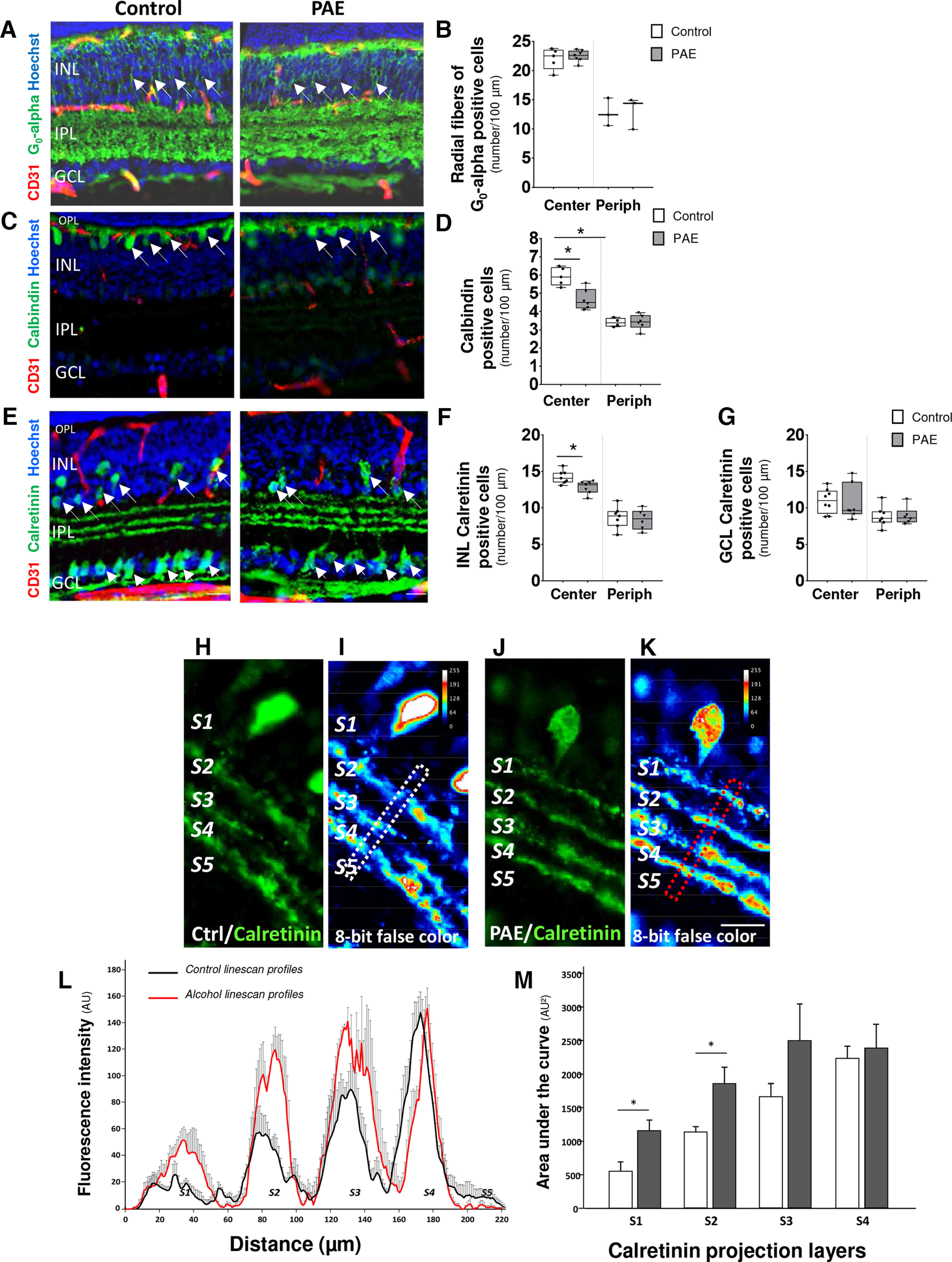
Effect of PAE on interneurons. ***A***, ***B***, Effect of PAE on bipolar cell layering. Triple fluorescent labeling visualizing bipolar cells, vessels, and nuclei labeled by G_0_-α, CD31 antibodies, and Hoechst in cross sections of control and PAE retinas. Arrows indicate radial fibers of bipolar cells. The scale bar represents 25 μm. In the central (Center) and the peripheral (Periph) parts of retinas, quantification of G_0_-α-positive vertical fibers per 100 μm (***B***) in the INL. ***C***, ***D***, Effect of PAE on calbindin-positive interneurons. Triple fluorescent labeling visualizing interneurons, vessels, and nuclei labeled by calbindin, CD31 antibodies, and Hoechst. In the central (Center) and the peripheral (Periph) parts of retinas, quantification of calbindin-positive cells per 100 μm in the OPL (***D***) in cross sections of control and PAE retinas. ***E–G***, Effect of PAE on calretinin-positive cell layering. High magnification of cross-section labeling using calretinin, PECAM/CD31 antibodies, and Hoechst in control and PAE mice. Calretinin-positive cells are visualized in the GCL (arrowheads) and INL (arrows). In the central (Center) and the peripheral (Periph) parts of retinas, measurement of calretinin-positive cells per 100 μm in the INL (***F***) and GCL (***G***), *p < 0.05; compared with the age-matched control group using the Mann–Whitney test, *n* = 5–7 pups per group. The scale bar represents 50 μm. Visualization of calretinin-positive cells at low magnification is shown in Extended Data Figure 4-1 and visualization of calretinin and calbindin-positive cells is shown in Extended Data Figure 4-2. ***H–K***, Visualization at high magnification of calretinin-positive dendritic projections in the IPL in the control (***H***) and PAE (***J***) groups. S1–S5 indicate the five parallel sublaminas of the IPL. The 8-bit false color encoding visualizes the relative immunolabeling intensities between sublaminas in the control (***H***) and PAE (***J***) groups. The dotted rectangles illustrate the MetaMorph scanline tool used to generate the intensity profiles. The scale bar represents 50 μm. ***L***, Visualization of mean intensity profiles associated with calretinin-positive projections in S1–S5 sublaminas of the IPL in the control (black curve) and PAE (red curve) groups. ***M***, Quantification of the areas under the curves in control (white bars) and PAE (black bars) P15 retinas. *p < 0.05 compared with the age-matched control group using the unpaired *t* test, *n* = 4 pups per group. GCL: ganglion cell layer; OPL: outer plexiform layer; INL: inner nuclear layer; IPL: inner plexiform layer; PAE: prenatal alcohol exposure.

Visualization of retinal interneurons, i.e., bipolar, horizontal, and amacrine cells was conducted using αG_0_, calbindin, and calretinin antibodies (Fig. 4), respectively. As expected, most cell bodies positive for all antibodies used were located in the INL. On sections passing through the center of the retina, intensely αG_0_-positive cells were identified at the outer border of the INL at P15 (Fig. 4*A*), indicating that the cell bodies of bipolar cells were correctly positioned in both control and PAE retinas. Quantification of radial bipolar fibers within the INL (Fig. 4*A*,*B*, arrows) did not reveal any significant differences at P15 (Fig. 4*B*).

10.1523/ENEURO.0295-22.2022.f4-1 Extended Data Figure 4-1 Effect of PAE on calretinin-positive interneurons. ***A***, ***B***, Triple fluorescent labeling visualizing interneurons, vessels and nuclei labeled by calretinin, CD31 antibodies, and Hoechst. Acquisitions were performed at P15 (***A***, ***B***) in the control (***A***) and PAE (***B***) groups. The scale bar represents 50 μm. GCL: ganglion cell layer; INL: inner nuclear layer; IPL: inner plexiform layer; ONL: outer nuclear layer; OPL: outer plexiform layer; PAE: prenatal alcohol exposure. Download Figure 4-1, TIF file.

10.1523/ENEURO.0295-22.2022.f4-2 Extended Data Figure 4-2 Co-visualization of calretinin and calbindin-positive interneurons in mouse retina at P15. ***A***, Triple fluorescent labeling visualizing calretinin (red), calbindin (green) and Hoechst (blue). The dotted square indicates the area visualized in *B-D*. Scale bars represent 100 μm. ***B***, Visualization at higher magnification of calretinin-positive neurons in the INL (arrow). ***C***, Visualization at higher magnification of calbindin-positive neurons in the INL (arrow). ***D***, Overlay of the two immunofluorescent signals. Note that in several neurons calretinin and calbindin immunoreactivities are co-localizing (arrow). Scale bars represent 50 μm. GCL: ganglion cell layer; INL: inner nuclear layer; ONL: outer nuclear layer. Download Figure 4-2, TIF file.

Regarding inhibitory interneurons, at P15, most calbindin immunoreactive cells from control retinas were localized in the OPL border of the INL, which corresponds to the final positioning of horizontal cells (Fig. 4*C*,*D*, arrows). Cell density was significantly higher in the central part of retina compared with the periphery and significantly decreased in the center of PAE retinas (*p* = 0.008, controls *n* = 5 and PAE *n* = 6, *U* = 1, Mann–Whitney test; Fig. 4*D*). In P15 control retinas, calretinin-expressing cells were detected in the INL and GCL (Fig. 4*E*, arrows). Quantification of cell density showed that these interneurons were at higher density in the central part of retina compared with the periphery and that PAE induced a significant decrease in calretinin-positive cells in the INL (*p* = 0.011, *n* = 7 for control and PAE groups, *U* = 5, Mann–Whitney test; Fig. 4*F*) without any effect on the cells positioned in the GCL (Fig. 4*G*).

Projections of amacrine cells in the IPL were also analyzed at P15. In the control retina, calretinin-positive projections were mainly localized in strata S2, S3, and S4 of the IPL at P15 (Fig. 4*H–K*). Intensity profiles using scanline acquisitions were performed. Quantitative analysis after false color visualization (Fig. 4*I*,*K*) and signal integration (Fig. 4*L*,*M*) revealed that in the control retinas, calretinin-positive projections were the lowest in strata S1 and S5 and the highest in stratum S4 (Fig. 4*L*, black curve; Extended Data Fig. 4-1*A*,*B*, arrow). PAE dramatically impaired the distribution pattern of calretinin-positive projections (ANOVA one-way (*F*_(7.24)_ = 6.317, *p* = 0.0003, *n* = 4; Fig. 4*H–M*). In particular, PAE significantly increased calretinin-positive projections in strata S1 (2-fold increase, *p* = 0.0028, *n* = 4 for two groups, Unpaired *t* test, *t* = 2.876) and S2 only (2.5-fold increase, *p* = 0.032; *n* = 4 for two groups, Unpaired *t* test, *t* = 2.766; Fig. 4*L*,*M*, red curve).

### Calretinin-positive cells are associated with perforating microvessels in the developing retina

**Figure 5.**
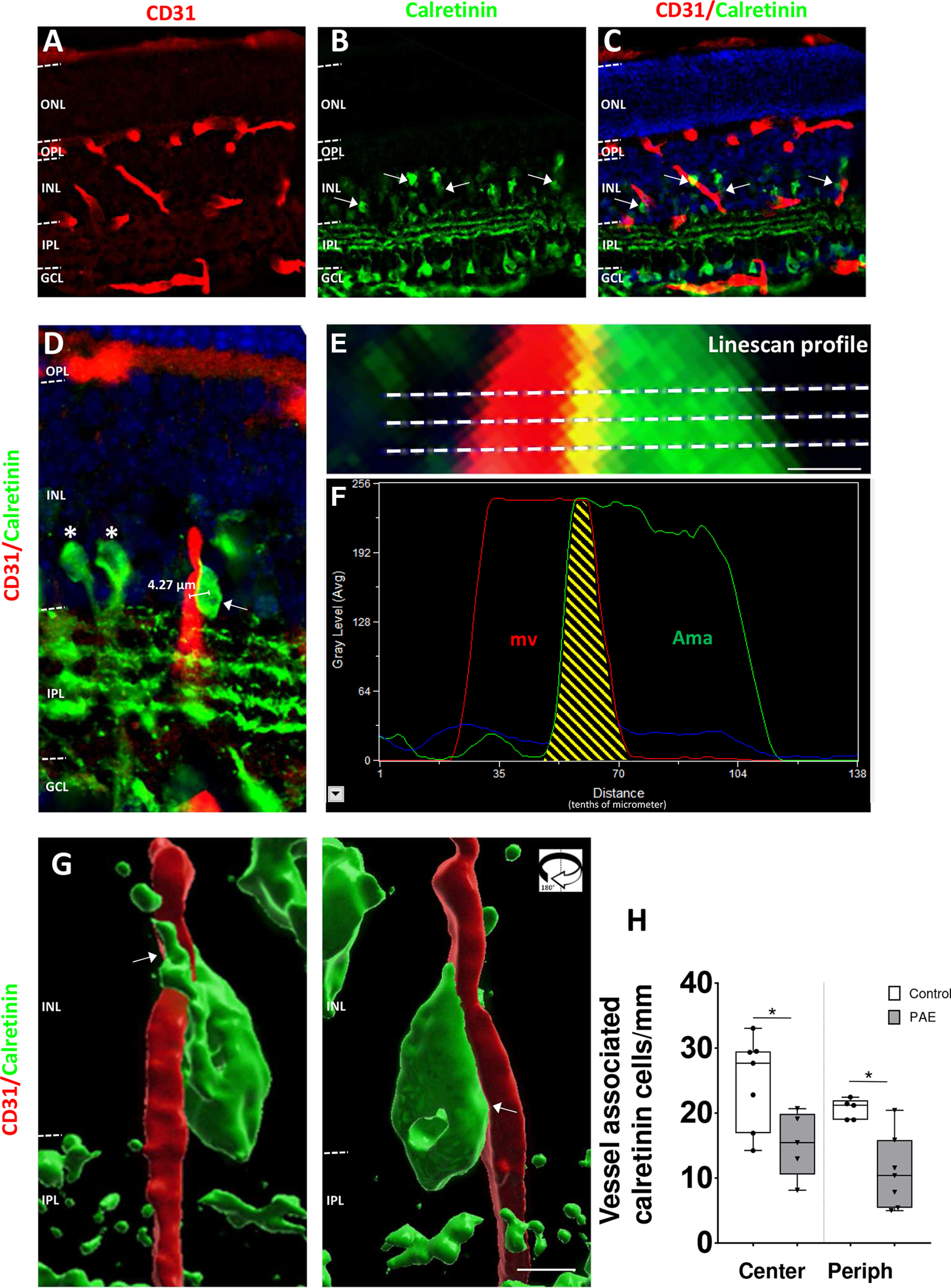
Identification of vessel-associated calretinin-positive cells. ***A–C***, Typical triple fluorescence acquisitions visualizing PECAM/CD31-positive (***A***), calretinin-positive (***B***), and Hoechst-labeled (***C***) cells in a postnatal days (P)15 PAE retina. The scale bar represents 30 μm. ***D*,** High-magnification detail visualizing a perforating microvessel (PECAM/CD31) and calretinin-positive cells in the INL of a P15 PAE retina. The arrow indicates a calretinin-positive cell in close interaction with a perforating microvessel. Stars indicate calretinin-positive cells presenting a morphotype of mature amacrine cells. Note that these cells are not vessel-associated. The scale bar represents 20 μm. ***E***, ***F***, Linescan profiles (***E***) and associated intensity curves (***F***) from a *Z* plane after confocal acquisition of the image presented in ***D***. Note the overlap (yellow area) of the PECAM/CD31 and calretinin intensity curves indicating cell proximity. The scale bar represents 1.5 μm. ***G***, IMARIS 3D map of the vessel-associated calretinin-positive cells presented in D. Scale bar represents 5 μm. ***H***, In the central (Center) and the peripheral (Periph) parts of retinas, measurement of calretinin-positive cells associated with vessels in the control (white boxes) and PAE (gray boxes) groups. *p < 0.05; compared with the age-matched control group using the Mann–Whitney test, *n* = 5–7 pups per group. GCL: ganglion cell layer; INL: inner nuclear layer; IPL: inner plexiform layer; ONL: outer nuclear layer; OPL: outer plexiform layer; PAE: prenatal alcohol exposure; RNL: retinal neuroblastic layer.

Since some GABAergic interneuron subpopulations migrate along radial microvessels in the developing neocortex (Léger et al., 2020a,b; Won et al., 2013), we explored whether such neurovascular interactions exist in the developing retina. Double immunolabeling experiments targeting calretinin and microvessels were performed at P15 (Fig. 5). Low magnification acquisitions revealed that calretinin-positive neurons were frequently localized in the vicinity of perforating microvessels (Fig. 5*C*, arrows). High magnification acquisitions revealed that these interneurons had the characteristic morphology of migrating cells: a fusiform cell body with a long primary dendrite oriented toward the outer part of the retina (Fig. 5*D*, arrow). In contrast, amacrine cells located in the INL and projecting into the IPL which had a morphology of mature neurons were not associated with microvessels (Fig. 5*D*, stars). Z-stack acquisitions were performed to characterize cell–cell interactions between calretinin-positive neurons and perforating microvessels (Fig. 5*E*,*F*). Calretinin-positive neurons were located at a mean distance of 6.32 ± 0.23 μm from the microvessels (*n* = 4; Fig. 5*D*). Analysis of the red (microvessels) and green (interneurons) linescan profiles revealed a thin overlap of the two signals supporting physical interactions between calretinin-positive cells and microvessels (Fig. 5*E*,*F*). 3D IMARIS maps made it possible to confirm the neurovascular interaction with a primary dendrite surrounding the microvesse (Fig. 5*G*). These results indicate that migrating immature interneurons are closely associated to perforating microvessels. Quantification revealed that PAE significantly reduced the number of calretinin-positive interneurons associated to vessel in the center and the peripheral part of retina (Center *p* = 0.048, controls *n* = 5 for control and *n* = 7 for PAE groups, *U* = 5, Mann–Whitney test; Periph: *p* = 0.01, controls *n* = 5 for control and *n* = 7 for PAE groups, *U* = 2, Mann–Whitney test; Fig. 5*H*).

### PAE prevents vessel association of calretininergic interneurons

**Figure 6.**
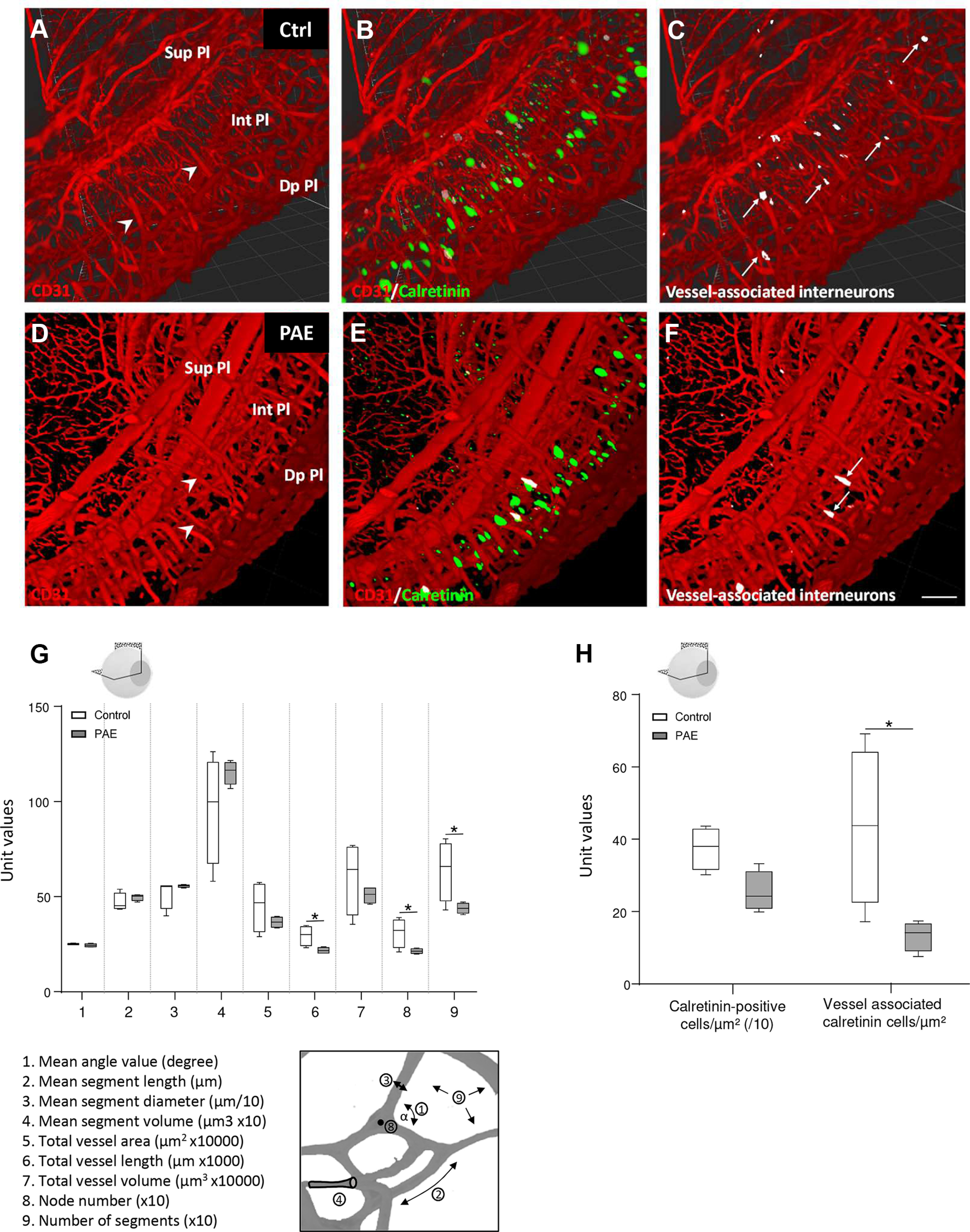
Effect of PAE on vessel-associated calretinin cells in cleared whole eyes from postnatal days (P)15 mice. ***A–F***, IMARIS 3D maps of PECAM/CD31-positive (red) and calretinin-positive (green) structures in cleared whole eyes from control (***A–C***) and PAE (***D–F***) P15 mice. 3D reconstruction was performed in a quarter of the cleared eye. Calretinin-positive cells associated with perforating microvessels are visualized in white. ***G***, Quantification of the effect of PAE on the whole retinal microvasculature. IMARIS analysis covered nine morphometric items. 1, mean angle value; 2, mean segment length; 3, mean segment diameter; 4, mean segment volume; 5, total vessel area; 6, total vessel length (*p* = 0.0148, *U* = 9), seven-total vessel volume; eight-node number (*p* = 0.007, *U* = 7); nine-number of segments (*p* = 0.007, *U* = 7). ***H***, Quantification of calretinin-positive cells and vessel-associated calretinin-positive cells in the control (white boxes) and PAE groups (gray boxes). *p < 0.05 compared with the age-matched control group using the Mann–Whitney test, *n* = 5 pups per group. Ctrl: Control; PAE: prenatal alcohol exposure.

To validate the neurovascular interactions between calretinin-positive interneurons and perforating microvessels with an integrated model, double immunostainings experiments were performed in cleared whole retinas from P15 mice using calretinin and CD31 antibodies (Fig. 6). 3D maps were generated to visualize the retinal microvasculature and, particularly the perforating microvessels (Fig. 6*A*,*D*, arrowheads). IMARIS analysis revealed that PAE did not modify the mean unitary angle values of branching vessels, mean segment length, mean vessel diameter or mean segment volume, suggesting that PAE did not modify the vascular network morphology (Fig. 6*G*, items 1–4). In contrast, PAE induced a substantial reduction in the number of nodes, number of segments, and total vessel length (Fig. 6*G*, items 6, 8, and 9), reminiscent of data obtained from whole-mount retinas and displaying a reduction in the mesh number and vessel network. Similarly, a 3D map of calretinin-positive interneurons was built, and cell quantification indicated that PAE tended to decrease the number of calretininergic interneurons, although this effect was not significant (Fig. 6*B*,*E*,*H*). In contrast, the overlay of CD31-immunoreactive and calretinin-immunoreactive 3D maps confirmed that several calretinin-positive interneurons were closely associated with perforating microvessels (Fig. 6*C*,*F*, arrows; Movie 1) and that PAE significantly reduced the density of calretinin-positive interneurons (*p* = 0.0159, controls *n* = 5 for control and PAE groups, *U* = 1, Mann–Whitney test) and the number of calretinin-positive interneurons associated to perforating vessel (*p* = 0.0159, controls *n* = 5 control and PAE groups, *U* = 1, Mann–Whitney test; Fig. 6*H*).

Movie 1. IMARIS 3D video of PECAM/CD31-positive (red), calretinin-positive (green), and Vessel-associated calretinin-positive (white) structures in cleared whole eyes from control P15 mice. 10.1523/ENEURO.0295-22.2022.video.1

### Normal positioning of calretininergic interneurons is disrupted in human FAS

**Figure 7.**
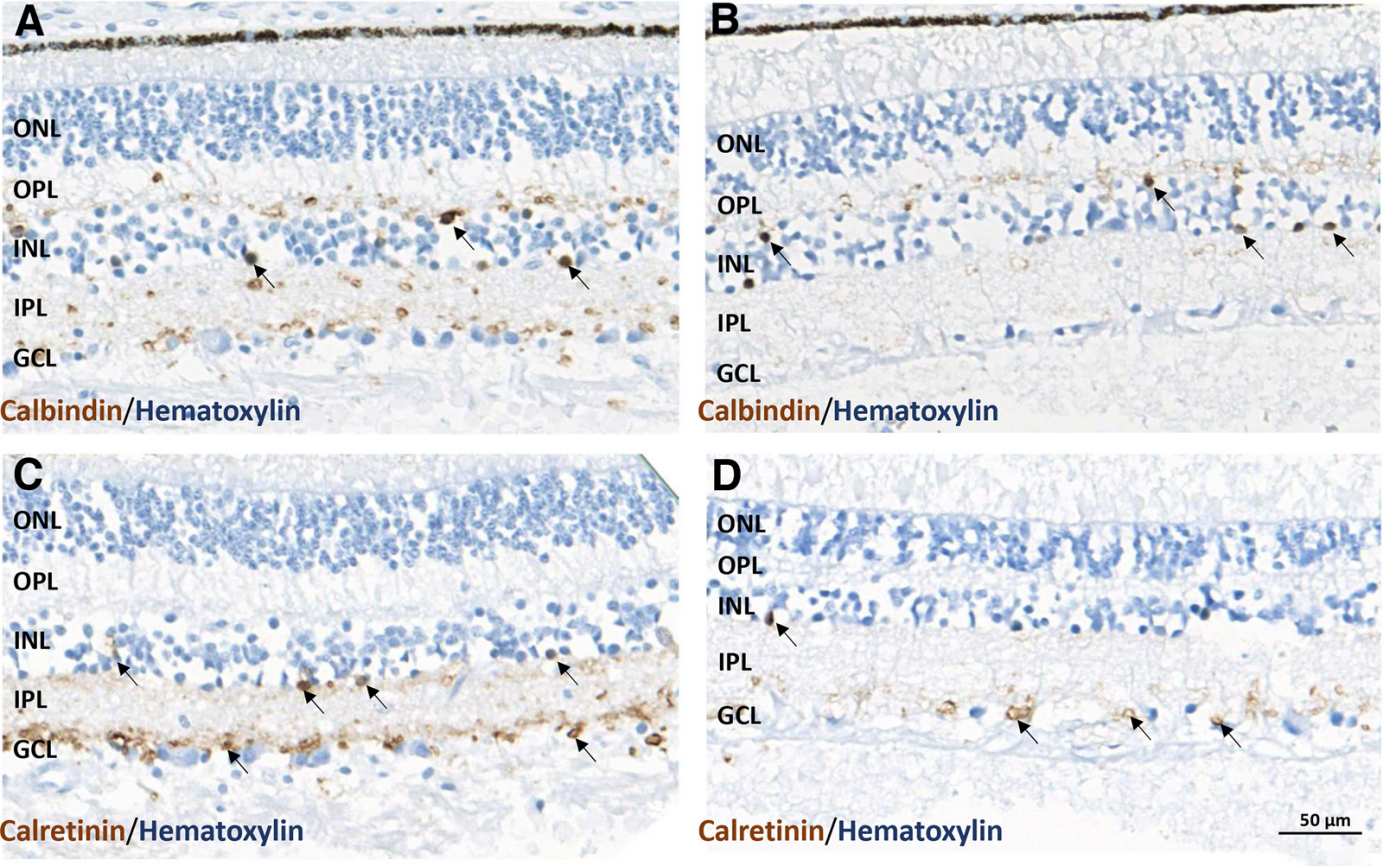
Calbindin and calretinin immunoreactivities in the retina of the FAS and control cases. ***A***, Visualization of calbindin-positive interneurons in the INL of the retina in the control case (arrows). ***B***, Compared with the FAS case (arrows). ***C***, Normal positioning of calretinin-positive interneurons in the INL of the human retina in the control case (arrows). ***D***, Contrasting with severe rarefaction of calretinin-positive interneurons in the INL of the FAS case but with a normal positioning in the GCL (arrows). The scale bar represents 50 μm. GCL: ganglion cell layer; INL: inner nuclear layer; IPL: inner plexiform layer; ONL: outer nuclear layer; OPL: outer plexiform layer.

The distribution profiles of calbindin-positive and calretinin-positive interneurons were analyzed in a three-month-old FAS infant and a five-month-old control infant (Fig. 7*A–D*). In the control and FAS retinas, calbindin-positive interneurons were localized in the INL (Fig. 7*A*, arrows, *B*, arrows). Quantification indicated that the density of calbindin-positive cells in the FAS retina was lower than that in the control one (Table 2). Regarding calretinin-positive interneurons, immunoreactive cells were localized in the INL and GCL in the control retina (Fig. 7*C*, arrows). In the FAS retina, calretinin-positive interneurons were scarce in the INL, and several positive cells were detected in the GCL (Fig. 7*D*, arrow). Quantification of immunoreactive cells showed that the INL/GCL ratio was lower in the FAS than in the control retina (Table 2).

**Table 2:** Distribution of calretinin and calbindin-positive neurons in retinas from five-month-old control and three-month-old alcohol-exposed neonates

Human case	Calbindin INL	Calretinin INL	Calretinin GCL	Calretinin INL/GCL ratio
				
Control retina (5 months old)	1.61 ± 0.17	0.75 ± 0.23	0.67 ± 0.24	1.36 ± 0.36
PAE retina (3 months old)	0.92 ± 0.14	0.39 ± 0.06	1.04 ± 0.29	0.40 ± 0.04

For each case, SEM illustrates the intrinsic dispersion of the interneuron densities.

## Discussion

This study provides the first evidence that; (1) PAE impairs both the development of the retinal microvasculature and neuronal organization, (2) in the developing retina, migrating calretinin-positive cells of the INL are associated with perforating microvessels, (3) PAE reduces the number of vessel-associated calretininergic interneurons, alters their positioning and disorganizes their dendritic projections in the IPL, and (4) the mispositioning of calretinin-positive cells observed in a murine FASD model is also observed in a FAS infant (visual abstract 1).

### Prenatal alcohol exposure affects retinal microvasculature development

Several studies performed in preclinical models of FASD and in FAS human brains have shown that PAE alters the cortical microvasculature (Jégou et al., 2012; Girault et al., 2017; Léger et al., 2020b) and that these angiogenic impairments result in neurovascular dysfunction (Lecuyer et al., 2017; Léger et al., 2020b). In the present study, we demonstrated using a murine model of FASD that PAE during the last gestational week delayed the postnatal development of the superficial vascular plexus. This effect was associated with a decrease in microvessel density and disorganization of this retinal microvascular plexus. Interestingly, PAE affected the central and peripheral parts of the retina differently. At P15, the density of microvessels was increased in the central part of the retina after PAE, while it was decreased at the periphery. Such results suggest an effect of PAE on vessel maturation. The development of the primary retinal vascular plexus proceeds in two steps: (1) formation of nascent microvessels by sprouting and (2) remodeling of the vascular plexus by pruning (Franco et al., 2015). Pruning is essential for the maturation of the vascular network and consists in regression or persistence of microvessels leading to a hierarchical organization of larger trunks and smaller branches to generate capillary-free zones around the major vessels (Fruttiger, 2007). Previous studies have already shown that ethanol is able to alter angiogenesis in the developing brain (Lecuyer et al., 2017; Li et al., 2021). The present data suggest that, in addition to angiogenesis, pruning could also be impaired.

In mice as well as in humans, as the superficial plexus is near completion, retinal vessels dive into the retina to form the deep vascular plexus at the base of the outer plexiform layer (Fruttiger, 2007; Selvam et al., 2018). The intermediate vascular network then forms and finalizes a well-organized network of three vascular plexuses (Sim and Fruttiger, 2013). The present data revealed that PAE induced a severe reduction in the number of perforating vessels and, although less pronounced, of the deep plexus, suggesting that PAE altered the development of all vascular structures. However, in contrast to clinical observations made in FAS children (Strömland, 1987), no tortuosity of the large vessels was found in our murine FASD model. One hypothesis could be raised to explain this discrepancy. In humans, tortuosity of the large vessels has been described in FAS children (Strömland and Pinazo-Durán, 2002), suggesting that alteration of large vessels would reflect severe exposition, such as facial dysmorphisms. Altogether, these data suggest that a fine analysis of the retinal microvasculature could provide new tools to detect impairments induced by moderate/short alcohol exposures.

### Calretinin-positive interneurons re-entering the INL are associated to vessels

In addition to microvascular defects, the present study reveals that PAE induced several neurodevelopmental abnormalities, such as a reduced retinal thickness and a decrease in cone, horizontal, and ganglion cell densities. Some of these alcohol-induced effects were previously reported in both human and animal models (Dursun et al., 2011; Deng et al., 2012; Muralidharan et al., 2015). For example, the offspring of chronically ethanol-fed rats presented a reduced eyeball weight, loss of ganglion cells, or reduced retinal thickness (Pinazo-Duran et al., 1993, 1997; Strömland and Pinazo-Durán, 2002). Concerning photoreceptor homeostasis, Katz and Fox reported, in a rat model, that alcohol exposure reduced rod sensitivity and dark adaptation (Katz and Fox, 1981). This effect was associated with a decrease in rhodopsin content. Our study highlighted that each subtype of photoreceptor was differentially impacted by PAE. We observed a specific decrease in the density of rods and opsin-B cones, whereas a slight increase in opsin-R/G cones was detected, indicating that dark adaptation and color visual functions are likely affected by PAE. These results supported that, as previously observed in FAS children, loss of visual function is associated with FASD. The relevance and applicability of these data to the diagnosis of FASD remain to be demonstrated, although they suggest that similar retinal alterations may occur in human FASD.

The present study provides new data regarding inhibitory interneurons, in particular concerning the positioning of calretinin-positive interneurons and their dendritic projections into the IPL which were evidently impaired by PAE, and these effects persisted in adult mice. Horizontal and amacrine cells are interneurons lying in the INL (Diamond, 2017). Horizontal cells are positioned at the most apical side, while amacrine cells reside at the most basal region of the INL. After genesis from neuroepithelial progenitors, the definite positioning of these neurons is a key event for appropriate laminar architecture and functional neuronal circuits within the visual system. Over the past decade, several studies over the past decade have provided evidence that horizontal and amacrine cells cover a substantial distance along the radial apico-basal direction of the retina before returning to the apical location where they later reside (Amini et al., 2018). First, amacrine and horizontal cell precursors migrate from the apical side using somal translocation, and second, they switch to a multipolar mode of migration to migrate back and re-enter deeper into the INL. Whereas amacrine cells move over short distances while translocating deeper into the INL (Chow et al., 2015), horizontal cells revert their trajectory toward the most-apical region of the INL (Edqvist and Hallböök, 2004; Poché et al., 2007). Mechanisms controlling the reverse migration of horizontal and amacrine cells by a multipolar mode are far from being understood. Interestingly, recent studies reported a multimodal migration mode of inhibitory neurons involving a close interaction with the microvasculature in the developing neocortex (Won et al., 2013; Léger et al., 2020a). GABAergic interneurons migrate first tangentially (Won et al., 2013) and, secondly, invade the neocortex using radial microvessels as guides (Léger et al., 2020a). In the present study, 3D map reconstructions from Z-stack acquisitions performed on flat-mount retina and whole cleared retinas showed that numerous calretinin-positive interneurons were closely associated to perforating vessels, as recently demonstrated in the developing neocortex (Léger et al., 2020a). These calretinin-positive interneurons had a single dendritic cone turned to the apical side of the INL and a dendrite wrapping perforating microvessels, a morphotype similarly to the migrating GABAergic interneurons entering the immature neocortex along radial microvessels (Léger et al., 2020a). Interestingly, PAE significantly reduced the density of calretinin-positive interneurons associated to vessels. Our original findings constitute the first evidence in favor of a vessel-associated migration of calretinin-positive neurons re-entering the INL. Whereas in mature retina, calretinin and calbindin are good markers of amacrine and horizontal cells, respectively, several studies showed a progressive segregation between these two markers that are co-expressed in developing retinas (Hendrickson et al., 2007; Fosser et al., 2013). Consisting with these data from the literature, double immunostaining experiments revealed several co-expressing interneurons in the INL (Extended Data Fig. 4-2). For this reason, the term calretinin-positive interneurons was used rather than amacrine cells. Among possible research avenues, it would be tempting to compare the migration pattern of amacrine versus horizontal cells by focusing on representative markers of the temporal patterning (Cherry et al., 2009).

Even if these data open new research avenues including functional studies, they support that PAE would impair a vessel-associated migration of interneurons as recently demonstrated in the developing cortex. In addition, because retinal interneurons are modulators of visual activity, the present data suggest that alcohol-induced dysfunction of inhibitory interneurons could contribute to the visual defects described in FAS children.

### Neocortex-retina transposition and clinical perspectives?

Recent preclinical and clinical discoveries regarding cortical neurodevelopment showed that *in utero* alcohol exposure impairs brain microvessel development (Léger et al., 2020a), the vessel-associated migration of GABAergic interneurons (Léger et al., 2020b) and accurate positioning within the different cortical layers (Marguet et al., 2020). The demonstration that the retina presents neurovascular similarities with the developing neocortex i.e., impaired development of the microvasculature, association of calretinin-positive interneurons with perforating radial vessels and mispositioning of amacrine cells in the INL of FASD mice and FAS infants, implies that careful analysis of the retinal microvasculature in human neonates may open new perspectives for an early diagnosis of *in utero* alcohol-exposed neonates. Fundus examination is a noninvasive and cost-effective tool, which, thanks to the advances in optical imaging technologies, allows for the visualization, quantification, and monitoring of retinal microvasculature (Rezar-Dreindl et al., 2021). Retinal examination could represent a new entry point to characterize alcohol-induced microvascular defects of the CNS. To reinforce such hypotheses, additional studies using *in vivo* optical imaging technologies such as OCT or electroretinograms should be performed in both FASD animal models and human neonates.

In conclusion, using a preclinical model of FASD, this study provides the first evidence that prenatal alcohol exposure induces neurovascular disorders in the developing retina. Alcohol exposure during pregnancy results in a severe disorganization of the microvascular plexuses associated with neuronal defects, including a mispositioning of interneurons. The fact that immature calretinin-expressing interneurons are vessel-associated provides the first neurovascular link between retinas neurovascular disorders and those described in the developing brain on PAE. These data contribute to a better understanding of the effects of alcohol on the developing visual system and reinforce that ophthalmological examination very likely becomes a promising tool for the early diagnosis of FASD.

## Synthesis

Reviewing Editor: Christopher Colwell, UCLA School of Medicine Decisions are customarily a result of the Reviewing Editor and the peer reviewers coming together and discussing their recommendations until a consensus is reached. When revisions are invited, a fact-based synthesis statement explaining their decision and outlining what is needed to prepare a revision will be listed below. The following reviewer(s) agreed to reveal their identity: Anna Klintsova. Note: If this manuscript was transferred from JNeurosci and a decision was made to accept the manuscript without peer review, a brief statement to this effect will instead be what is listed below.

Both reviewers and myself found that your work was intellectually interesting and technically strong. The reviewers made a few suggestions designed to improve the ms. Please address these concerns and re-submit.

Introduction:

Line 77 - in the sentence “Prenatal alcohol exposure (PAE) is a main cause of acquired mental retardation worldwide...” (line 77) please replace “mental retardation” with ......

Line 80 - “youth in the general population is estimated to be 7.7 per 1000 people (Popova et al., 2017)” -- the epidemiological data presented seems to be quite low

Materials and Methods:

Line 131-137 - How many pregnant dams (total) were used in the study? How many litters and pups? If the pups in the litters were culled to maintain uniform number of pups per litter this needs to be stated.

Line 140 - The volume of injections should be stated.

Line 145 - No details about immunocytochemical staining are provided, please include the antibodies concentrations here.

Line 154-155 - The details of the retina image acquisition should be described in more details.

Line 173-175 - Please clarify the sentence “Ice-cooled NEG-50TM tissue blocks were sectioned (Leica RM2235 Microtome using S35-PFM feather microtome blades) to generate serial sections (20 μm) using a cryostat (Leica CM1950, Nanterre, France).” The way it is written one has an impression that sectioning was done twice, first with microtome then with the cryostat.

Line 178 - Please, state the antibodies that were used to label the cells on the cross section of the retina in the text (in addition to the reference to Table 1). Concentrations for each and every primary and secondary antibody should be included.

Line 180 - the authors wrote “... incubation buffer containing the appropriate secondary antibody.” However, it is impossible to know what pairs of primary-secondary antibodies were used in the study.

Line 194-195 - Please, clarify what are the four ROI in the retina where the quantifications were performed. How was the number of animals per group (n=4) determined?

In Figure 4C, there seemed to also have calbindin-positive cells colocalized CD31-positive vessels. As horizontal cells are localized right next to the deep vasculature and reduced horizontal cells were found in both mouse PAE and human PAE retinas, were there any association between horizontal cells with perforating microvessels?

2. Amacrine cells at INL are closer to the intermediate vascular plexus, any change in this vascular layer in the later time point?

3. As there were different responses in the central and peripheral retina areas (Figure 2B), were there any differences in neuronal cells at different regions? Also vessel-associated amacrine cells?

4. Were the authors able to stain vessel markers with calbindin and calretinin antibodies in human retina? Any differences between PAE and control? Any correlation between vessels and neurons?
