# Supplementary figures and images for: In Utero Alcohol Exposure Impairs Retinal Angiogenesis and the Microvessel-Associated Positioning of Calretinin Interneurons
Source: eNeuro. 2023 Apr 25;10(4):ENEURO.0295-22.2022. doi: 10.1523/ENEURO.0295-22.2022 (PMC10135090; doi:10.1523/ENEURO.0295-22.2022)

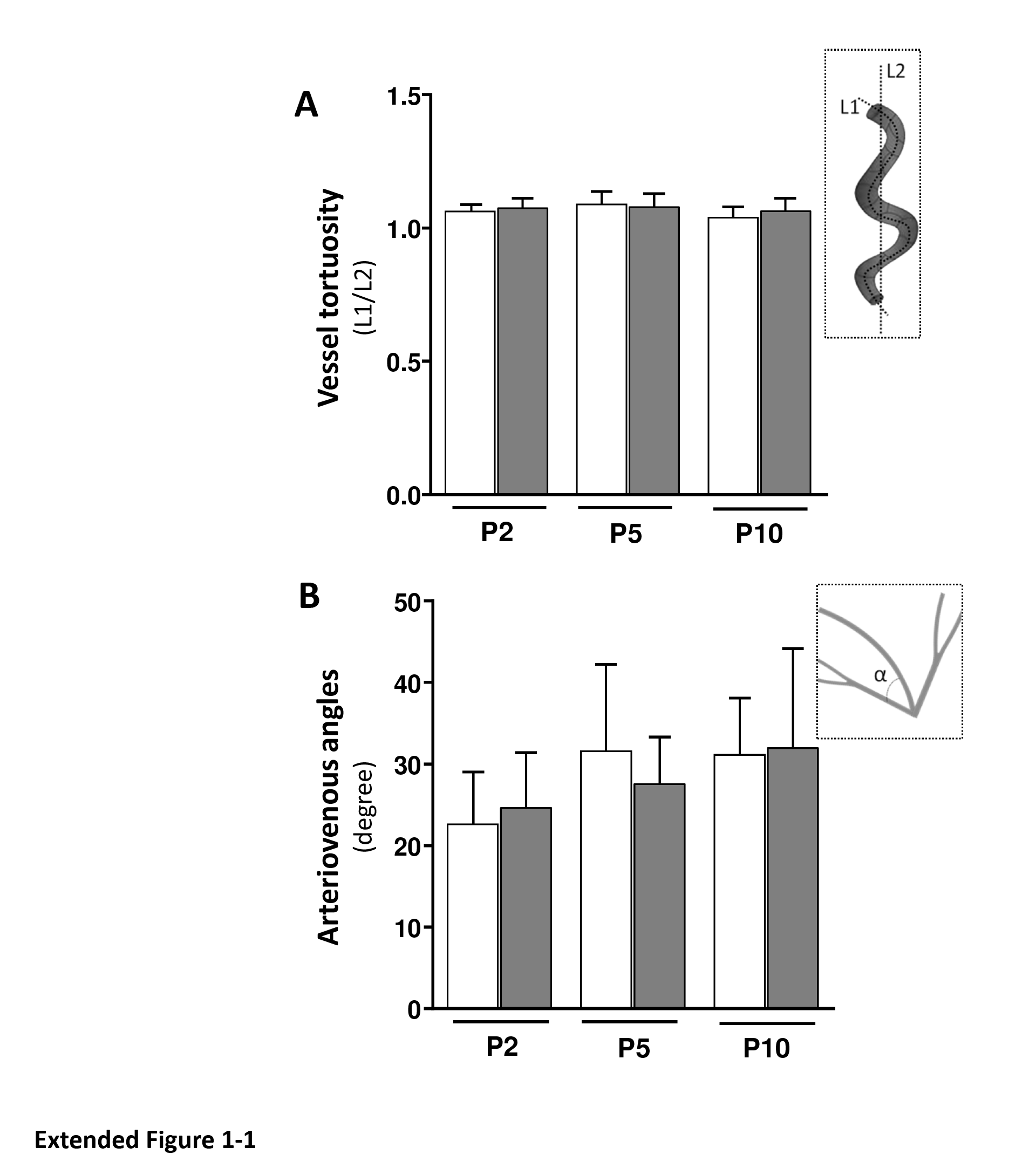

Supplement: Extended Data Figure 1-1 — Effect of PAE on vessel tortuosity and arteriovenous angles. Whole-mount retinas from postnatal day (P)2, P5, and P10 mice were immunostained with CD31 antibody to visualize microvessels. A, Quantification of vessel tortuosity in the control and PAE groups. The inset illustrates how the ratio L2 (measured vessel length)/L1 (theoretical straight vessel) was used as an index of tortuosity. B, Quantification of the arteriovenous angles in the control and PAE groups. The inset illustrates how the angle value was calculated between two large vessels originating from the optic nerve. Download Figure 1-1, TIF file. [file enu-eN-NWR-0295-22-s03.tif]

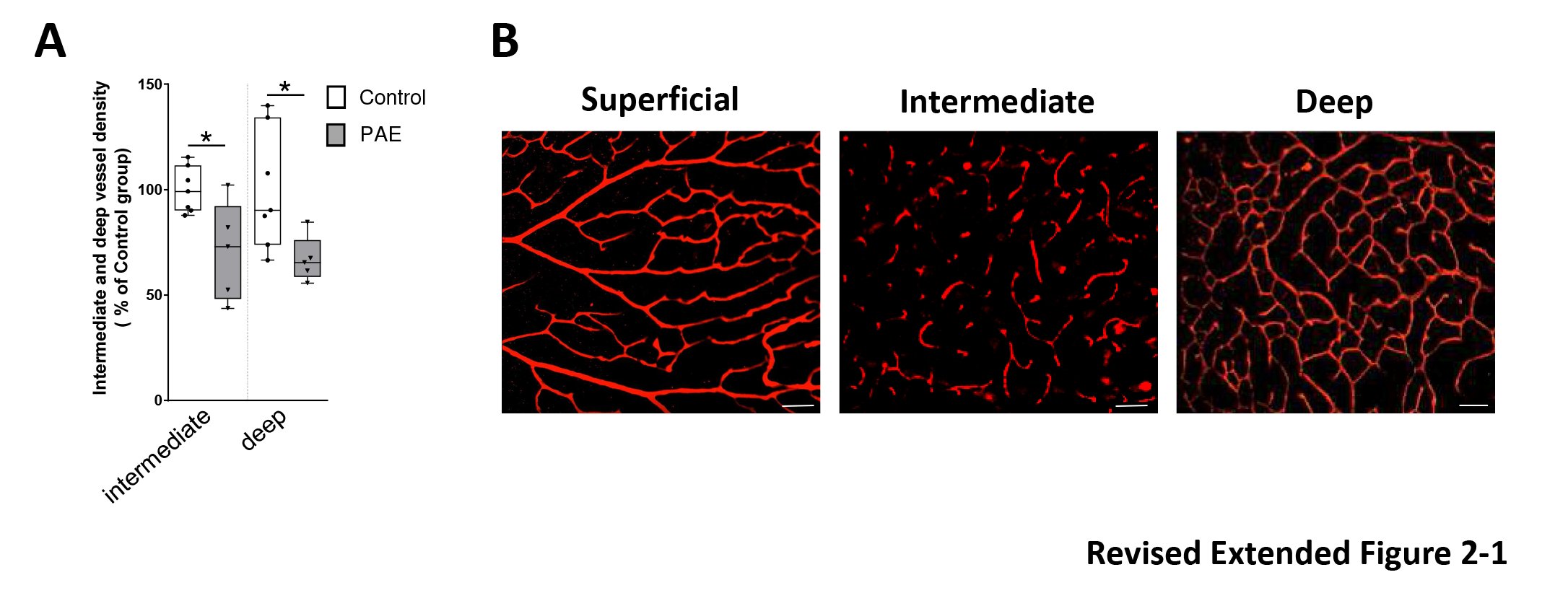

Supplement: Extended Data Figure 2-1 — Superficial, intermediate, and deep vascular plexuses in retina of P15 mice. Retinas from postnatal day (P)15 mice were immunostained with CD31 antibody to visualize microvessels. A, Quantification of vessel density of the intermediate and deep vascular plexus from control (white boxes) and PAE (grey boxes) groups in the center of retinas. *p < 0.05 compared with the age-matched control group. Mann–Whitney test, n = 5–7 pups per group. B, Visualization of the superficial, intermediate, and deep vascular plexus in the center part of retinas by confocal microscopy. Scale bars represent 50 μm. Download Figure 2-1, TIF file. [file enu-eN-NWR-0295-22-s04.tif]

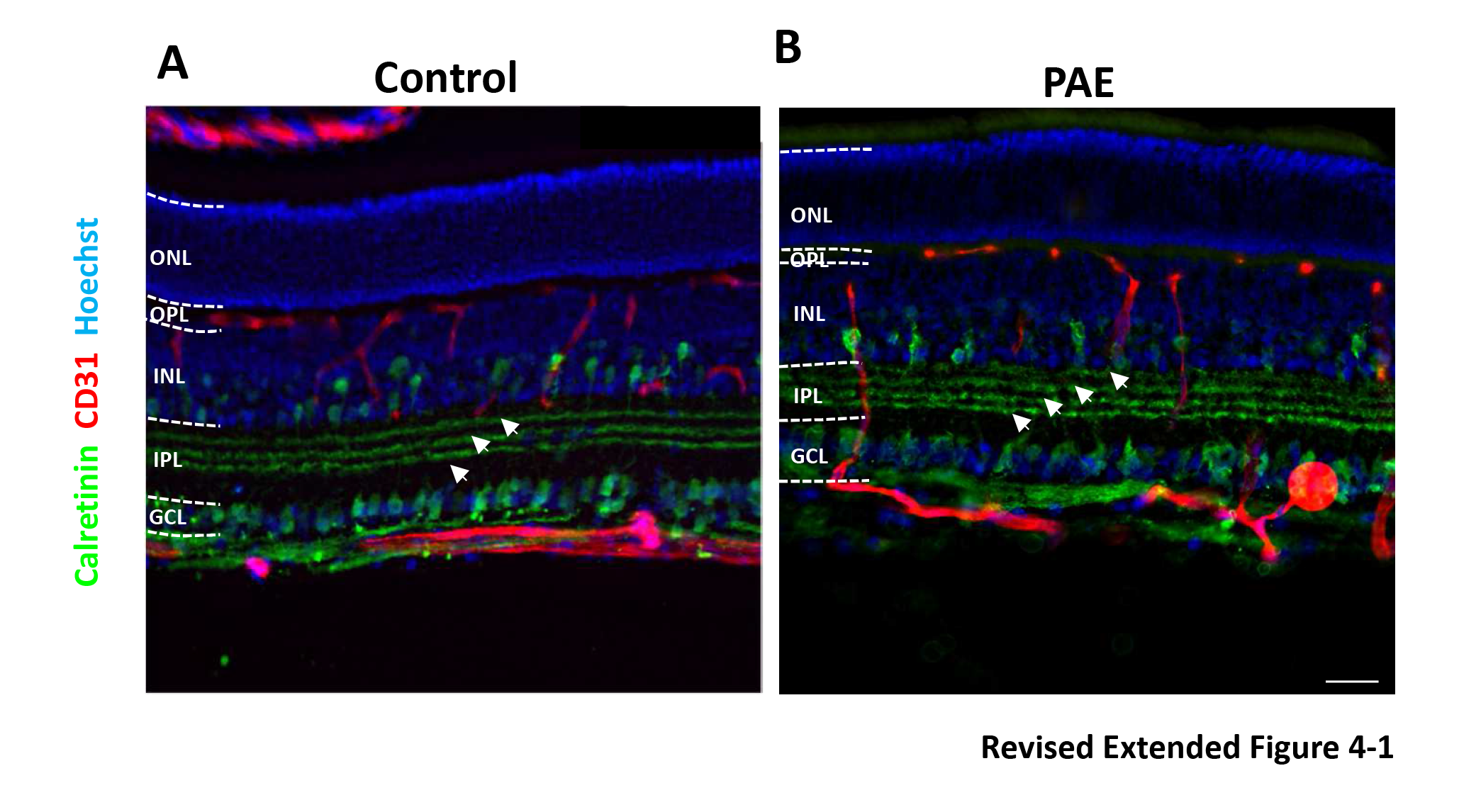

Supplement: Extended Data Figure 4-1 — Effect of PAE on calretinin-positive interneurons. A, B, Triple fluorescent labeling visualizing interneurons, vessels and nuclei labeled by calretinin, CD31 antibodies, and Hoechst. Acquisitions were performed at P15 (A, B) in the control (A) and PAE (B) groups. The scale bar represents 50 μm. GCL: ganglion cell layer; INL: inner nuclear layer; IPL: inner plexiform layer; ONL: outer nuclear layer; OPL: outer plexiform layer; PAE: prenatal alcohol exposure. Download Figure 4-1, TIF file. [file enu-eN-NWR-0295-22-s05.tif]

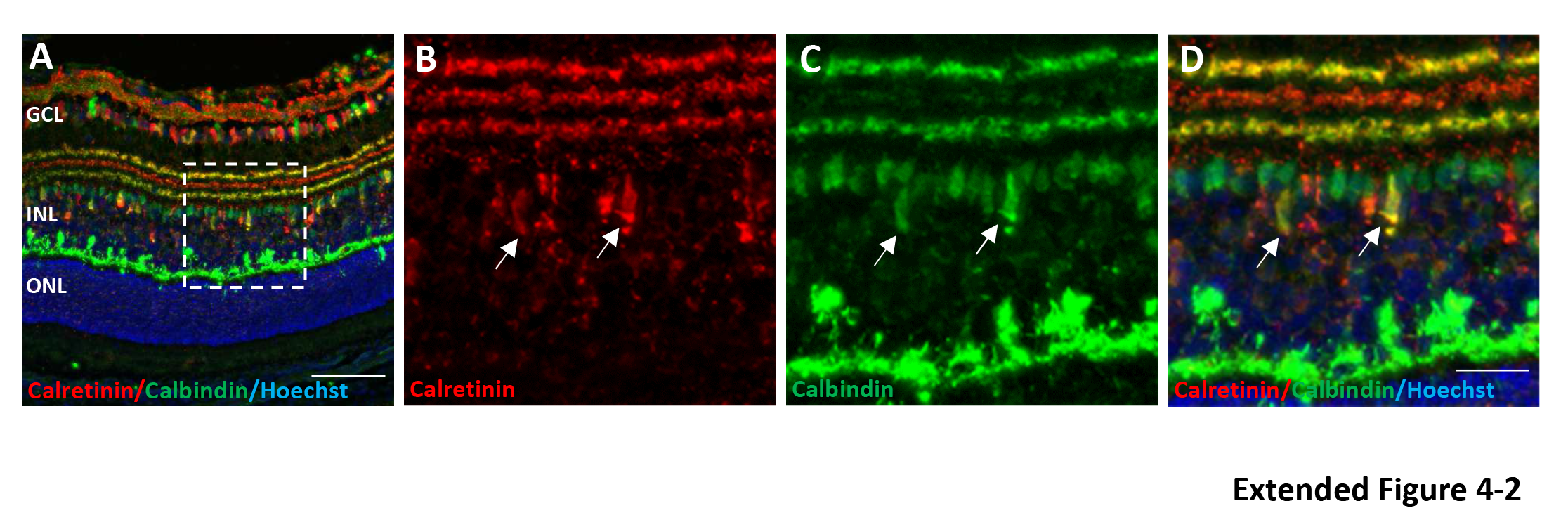

Supplement: Extended Data Figure 4-2 — Co-visualization of calretinin and calbindin-positive interneurons in mouse retina at P15. A, Triple fluorescent labeling visualizing calretinin (red), calbindin (green) and Hoechst (blue). The dotted square indicates the area visualized in B-D. Scale bars represent 100 μm. B, Visualization at higher magnification of calretinin-positive neurons in the INL (arrow). C, Visualization at higher magnification of calbindin-positive neurons in the INL (arrow). D, Overlay of the two immunofluorescent signals. Note that in several neurons calretinin and calbindin immunoreactivities are co-localizing (arrow). Scale bars represent 50 μm. GCL: ganglion cell layer; INL: inner nuclear layer; ONL: outer nuclear layer. Download Figure 4-2, TIF file. [file enu-eN-NWR-0295-22-s06.tif]
